# Nutrients and Pathways that Regulate Health Span and Life Span

**DOI:** 10.3390/geriatrics5040095

**Published:** 2020-11-19

**Authors:** Carla Pignatti, Stefania D’Adamo, Claudio Stefanelli, Flavio Flamigni, Silvia Cetrullo

**Affiliations:** 1 Department of Biomedical and Neuromotor Sciences, Alma Mater Studiorum, University of Bologna, 40126 Bologna, Italy; carla.pignatti@unibo.it (C.P.); flavio.flamigni@unibo.it (F.F.); 2 Department of Medical and Surgical Sciences, Alma Mater Studiorum, University of Bologna, 40136 Bologna, Italy; stefania.dadamo2@unibo.it; 3 Laboratory of Immunorheumatology and Tissue Regeneration, IRCCS Istituto Ortopedico Rizzoli, 40136 Bologna, Italy; 4 Department for Life Quality Studies, Alma Mater Studiorum, University of Bologna, 47921 Rimini, Italy; claudio.stefanelli@unibo.it

**Keywords:** nutrients, nutrient-sensing pathways, health span, life span, aging

## Abstract

Both life span and health span are influenced by genetic, environmental and lifestyle factors. With the genetic influence on human life span estimated to be about 20–25%, epigenetic changes play an important role in modulating individual health status and aging. Thus, a main part of life expectance and healthy aging is determined by dietary habits and nutritional factors. Excessive or restricted food consumption have direct effects on health status. Moreover, some dietary interventions including a reduced intake of dietary calories without malnutrition, or a restriction of specific dietary component may promote health benefits and decrease the incidence of aging-related comorbidities, thus representing intriguing potential approaches to improve healthy aging. However, the relationship between nutrition, health and aging is still not fully understood as well as the mechanisms by which nutrients and nutritional status may affect health span and longevity in model organisms. The broad effect of different nutritional conditions on health span and longevity occurs through multiple mechanisms that involve evolutionary conserved nutrient-sensing pathways in tissues and organs. These pathways interacting each other include the evolutionary conserved key regulators mammalian target of rapamycin, AMP-activated protein kinase, insulin/insulin-like growth factor 1 pathway and sirtuins. In this review we provide a summary of the main molecular mechanisms by which different nutritional conditions, i.e., specific nutrient abundance or restriction, may affect health span and life span.

## 1. Introduction

In humans the life span is dependent on genetic, environmental and lifestyle factors. The genetic components contribute for about 20–25%, while some components of lifestyle seem to play major roles [1,2]. Recent advances in the field of gerontology are showing that aging should be viewed as adaptive and amenable to interventions aimed at extending health span and life span. Over the last several decades, improvements of several lifestyle components such as nutrition, and hygiene education, together with medical advances and therapy have led to a significant increase in life expectancy. However, longer life expectancy can also lead to an increase of the number of people suffering from age-related diseases and age represents the main risk factor for all major life-threatening disorders. The fact that health span is not growing in the same way as life span is a source of great concern and it has raised interest among the scientific and medical community to study and elaborate strategies to improve health span. We highlight the importance of human studies to better understand interventions that could counteract the functional decline of tissues and organs and prevent the accumulation of molecular damage leading to multiple chronic diseases. Interestingly, the most effective interventions to improve healthy senescence to date converge on only a few cellular and biochemical processes, in particular nutrient signaling, mitochondrial efficiency, proteostasis, autophagy, microbiota modulation. The role of the genotype in aging and longevity is an important issue that we do not have the ability to favorably modify. However, growing evidence has shown that epigenetic changes could interfere with the genetic profile to deeply affect health span and, in some situations, to be even more important than the genetic profile. Indeed, alterations in DNA methylation, post-translational modification of histones and changes in the organization of chromatin have been demonstrated to influence health span and life span in several animal models (invertebrate organisms and vertebrate models, mostly rodents) [3].

Epidemiological, clinical and experimental studies actually showed that what we eat and how much we consume contributes to determine our health span [1]. Recently Longo hypothesized the existence of multiple “longevity programs” which are selected according to the availability of nutrients and that the key event for increased health span is the activation of regenerative processes that lead to “rejuvenation” also independently of aging rate [4]. The anabolic processes such as growth, reproduction and nutrient storage are promoted by the excess of nutrients, whereas nutrient limitation stimulates catabolism in order to serve energy and essential functions. Such pathways, which interact with each other, include the evolutionarily conserved key regulators mammalian target of rapamycin (mTOR), insulin and insulin-like growth factor 1 (IGF1) pathways, AMP-activated protein kinase (AMPK), sirtuins (SIRTs) and fibroblast growth factor 21 (FGF21) pathways. Their interrelations are presently under continuous and widespread investigation, and the complex network by which the nutrient signaling pathways mediate the effects of nutrients or various feeding regimens remains to be fully understood.

## 2. Nutrient Sensors that Potentially Affect Health Span

### 2.1. Sirtuin 1 (SIRT1)

SIRTs are crucial nutrient sensors extensively studied in the last decade because of their pleiotropic functions potentially affecting health span. In mammals, the SIRT family includes seven members (SIRT1-7) differentially located in cell compartments and exerting various roles. SIRTs affect the activity of proteins implicated in metabolism, oxidative stress, cell survival, autophagy, with important consequences on aging. Among SIRTs, SIRT1 is the best described in literature. This enzyme, like other components of the family, is a deacetylase and depends on NAD^+^ for its activity. Since this molecule accumulates in the cell typically during fasting or exercise, SIRT1 results to be activated in these situations of low energy levels [5]. Decreased NAD^+^ levels with aging in part explains the decrease of SIRT1 activity in elderly people. Therefore, this enzyme has been included among the most promising nutritional biomarkers [6]. SIRT1 deacetylase activity influences the function of many proteins implicated in cell metabolism. In this regard it has been shown that almost all enzymes of anabolic and catabolic pathways are highly acetylated [7]. Thus, SIRT1 activity may impact gluconeogenesis, glycolysis, fatty acid oxidation, tricarboxylic acid (TCA) cycle and oxidative phosphorylation. SIRT1 also deacetylates many transcription factors that control the alternative for the cell between an oxidative and an anabolic strategy. In liver SIRT1 deacetylates the transcription factor sterol regulatory element-binding protein 1c (SREPB1c), thus reducing its affinity for promoters of the lipogenic targets genes [8]. In adipocytes, SIRT1 favors corepressor efficiency on peroxisome proliferator-activated receptor γ (PPARγ) reducing adipogenesis. These effects increase fat mobilization instead of storage and induce favorable cellular and health changes [9]. SIRT1 expression increases during energy starvation in both mice and humans and declines under high fat diet or obesity. Studies in mice have also shown that SIRT1 overexpression conveys similar beneficial health effects as low calorie diets and protects various markers of health upon diet-, injury- and disease-related stressors [10,11].

SIRT1 has been extensively implicated in the prolongevity effect of caloric restriction (CR) in model organisms. Moreover, this enzyme represents a target in the mechanism of action of bioactive and health-promoting compounds, such as resveratrol and other polyphenols, also called CR mimetics (CRMs) [12] SIRTs have been extensively implicated in several age-related degenerative diseases, such as cancer, diabetes, cardiovascular disease and neurodegenerative disorders. These enzymes may exert neuroprotective effects and counteract some kinds of tumors, also by stimulating autophagy, a process of cellular “self-digestion”, an efficient way of biochemical recycling needed for maintaining cellular homeostasis and influence health span [13,14]. Both SIRT1 and SIRT3 activate autophagic machinery by deacetylation of key autophagic components [14]. Activation of autophagy can consequently activate mitophagy, leading to selective clearance of damaged mitochondria. In mitochondria, SIRT3 also deacetylates forkhead box O transcription factors (FOXOs) and mitochondrial superoxide dismutase (SOD2), resulting in greater respiratory efficiency, apoptotic resistance and protection from reactive oxygen species [15,16,17,18].

Stimulation of autophagy by SIRTs may be particularly relevant in elderly people as during aging a reduction in proteolytic activity and autophagic function has been reported, with a consequent accumulation of damaged proteins [13,19]. Reduced SIRT activity in aging has been in part related to decreased availability of NAD^+^ for deacetylase reactions due to higher activation of poly-[ADP-ribose] polymerase 1 (PARP1) in elderly people. This enzyme catalyzes ADP-ribosylation reactions and also needs NAD^+^ as a cosubstrate. PARP1 plays a crucial role in mechanisms of DNA repair. It has been demonstrated that the inhibition of PARP1 affects SIRT1 activity and oxidative metabolism by raising NAD^+^ levels [20]. Recently the relation between SIRT1 and autophagy has been further studied and a more complicated picture has been revealed. Indeed, new evidence reported that autophagy may downregulate SIRT1 contributing to its loss in senescence and ageing in several tissues related to the immune and hematopoietic system [21].

### 2.2. AMP-Activated Protein Kinase (AMPK)

AMPK is a conserved, energy-sensing serine/threonine kinase which is activated in case of low cellular energy levels resulting in increased levels of AMP. Indeed, the catalytic subunit of this enzyme (α) is controlled by the regulatory subunits β and γ, responding to the direct interaction with AMP and ADP and to the upstream kinases liver kinase B1 (LKB1) or calcium/calmodulin-dependent protein kinase kinase 2 (CaMKK2) [22].

AMPK stimulates catabolic processes that produce energy. Indeed, this enzyme is able to activate the uptake and the utilization of glucose and fatty acids, mitochondrial biogenesis and autophagy. At the same time the activation of AMPK inhibits by phosphorylation acetyl-CoA carboxylases (ACCs), glycerol phosphate acyl-transferases (GPATs), 3-hydroxyl-3-methylglutaryl CoA reductase (HMGCR) and glycogen synthase, thus repressing anabolic processes like fatty acid, triglyceride, cholesterol and glycogen biosynthesis [23,24]. Several transcription factors, like carbohydrate-responsive element-binding protein (ChREBP) and SREBP1c, are inhibited in liver by AMPK, which contributes to reprogram cell metabolism [25]. AMPK also inhibits a pro-inflammatory transcription factor, nuclear factor kB (NF-kB), by phosphorylating FOXO [26]. Conversely, AMPK increases the activity of the deacetylase SIRT1 and of the transcriptional coactivator peroxisome proliferator-activated receptor gamma coactivator 1α (PGC1α) [24]. AMPK has been considered a “pro-longevity kinase”, since its activation would be sufficient to extend lifespan in model organisms [27].

Activation of AMPK results in improvement of insulin sensitivity, fatty acid metabolism, mitochondrial performance and, in model organisms, this enhanced longevity [27,28]. AMPK appears to be beneficial for health, in particular in the context of obesity, nonalcoholic fatty acid disease (NAFLD), atherosclerosis and diabetes; therefore, therapeutic strategies aimed at activating AMPK remain promising for the treatment of metabolic diseases [28]. Activators of AMPK are many natural compounds, generally known as nutraceuticals, such as resveratrol, genistein, gallic acid, betaine. Additionally, some known small molecules are direct AMPK agonists, such as aspirin, rapamycin and metformin [27]. Of special relevance is that the antidiabetic drug metformin is a potent inducer of AMPK and has been suggested to be also a geroprotective agent. Metformin has been reported to reduce the risk of several aging-associated pathological conditions, including cardio-metabolic and neurodegenerative disorders, frailty and cancer in humans [29].

### 2.3. Forkhead Box O transcription Factors (FOXOs)

FOXO family transcription factors are central regulators of metabolic homeostasis, redox balance and stress response. In humans, the FOXO family comprises FOXO1, FOXO3, FOXO4 and FOXO6. Most FOXO genes are known to be implicated in human longevity [30]. In particular, the association of FOXO3 with human longevity has been evidenced by many research groups on different long-lived subjects [31,32].

FOXO functions are critical for coordinating a response to environmental fluctuations in order to maintain cellular homeostasis and support healthy aging [32]. FOXOs respond to a wide range of stimuli, including growth factors, hormones, oxidative and genotoxic stress and low nutrient availability. Downstream functions of FOXOs are conserved across species and include regulation of a variety of cellular processes and several longevity routes, such as autophagy, nutrient signaling, stress resistance, cell cycle arrest, suppression of inflammation and antioxidant activity (for a review: [30,31,33]). In the liver, low nutrient status and thus low levels of insulin signaling activate FOXOs to restore glucose levels via glycogenolysis and gluconeogenesis. Then, FOXO delivers a metabolic shift from glucose to fatty acid oxidation and enhances mitochondrial biogenesis. In human skeletal muscle the activation of FOXO3 by CR promotes upregulation of several anti-aging genes, including those responsible for antioxidant enzymes, DNA repair and autophagy [34].

FOXO activity is tightly regulated at the post-translational level in all species. FOXOs receive inputs from various signaling pathways in the form of covalent modifications, including phosphorylation, acetylation, methylation and ubiquitination, as well as of protein–protein interactions. FOXO3 in particular is activated through phosphorylation and deacetylation. Insulin signals through phosphoinositide 3-kinase (PI3K)-AKT phosphorylates FOXO proteins thus triggering their inactivation and nuclear exclusion. Phosphatase and tensin homolog (PTEN) inhibit the activation of PI3K thus promoting FOXO activation and nuclear localization. AMPK directly phosphorylates FOXO3 at six serine/threonine residues that are distinct from the AKT phosphosites and this event promotes interaction between cofactors and FOXO3 to affect specific target genes. Fasting conditions can up-regulate SIRT1, which deacetylases and activates FOXOs in the liver. This cellular context involves dual regulation: low insulin levels drive FOXOs into the nucleus, which are then further activated through deacetylation by SIRT1.

Although most studies have focused on post-translational regulation of FOXO factors, several groups have reported post-transcriptional regulation by microRNAs (miRNAs) [35]. Indeed, many miRNAs have been found to directly modulate FOXO transcript stability or translation. Moreover, this post-transcriptional regulation can also occur by RNA-binding proteins. These proteins act as mRNA-stabilizing, or regulators of mRNA splicing, transport and translation. Thus, the resulting picture of the post-transcriptional regulation of FOXO activity is quite complex and seems to respond to stress stimuli to promote adaptation in a variety of cell types and tissues and under diverse physiological and pathological conditions, including oxidative stress, cancer and age-related diseases.

### 2.4. Fibroblast Growth Factor 21 (FGF21)

FGF21 is an important mediator with a critical role in the transition from fasting to refeeding status. This molecule is mainly synthesized by the liver, which, during fasting, secretes it to coordinate the metabolic response. FGF21 activates fatty acid oxidation and ketogenesis in liver and, specifically in humans, this hepatokine is involved in the late adaptive response to fasting (7–10 days). In this condition, FGF21 induces the expression of PGC1α and late hepatic gluconeogenesis, but not glycogenolysis in order to spare hepatic residual glycogen reserves. Moreover, in this situation of low energy, FGF21 induces lipolysis and the production of adiponectin in adipose tissue and promotes AMPK-SIRT-PGC1α network. Conversely, FGF21 has an inhibitory effect on insulin/IGF1 signaling (IIS) pathway [36].

Interestingly, FGF21 seems to mimic CR, having a similar effect on gene expression in liver. Thus, it is conceivable that it has a potential role as a factor promoting life span, such as CR [37]. Some data suggested that the production of FGF21 is dependent on protein restriction, and in particular dependent on the low level of methionine intake [38,39]. Moreover, low protein-rich carbohydrate diets seem to be more effective in the stimulation of FGF21 production [40].

Adipose tissue is an additional production site of FGF21, however in this case it acts just locally, and its role is in close connection with the refeeding. Indeed, when there is the transition from late fasting (during which FGF21 by the liver operates) to refeeding (when liver stops to synthetize it), adipose tissue produces FGF21 in order to promote insulin-stimulated glucose uptake and counteract insulin insensitivity induced by the released fatty acids during fasting [41]. FGF21 also plays a role in the activation of brown adipose cells and in the “browning” mechanism of white adipocytes. In light of this, FGF21 receptor agonists are emerging as therapeutic drugs for the treatment of obesity-related diseases [42,43].

Recently, FGF21 as well as the fibroblast growth factor 19 have been involved in the signaling pathway that control muscle mass [44]. In normal conditions basal expression of FGF21 in muscle is low, however different physiological and pathological conditions such as exercise, fasting condition or mitochondrial stress may increase its level [45]. FGF21 has been reported to control muscle mass through the regulation of anabolic/catabolic balance and mitophagy [46]. Moreover, circulating FGF21 levels positively correlate with aging [47] and age-related sarcopenia [48]. This increase may also be a compensatory response to mitochondrial dysfunction to counteract energy insufficiency [49,50]. However, the direct contribution of this factor to muscle dysfunction related to aging has not been investigated yet.

### 2.5. Insulin-Like Growth Factor 1 (IGF1) and Growth Hormone (GH)

Components of IIS pathway are hormones, such as growth hormone (GH), insulin and IGF1, their specific receptors, proteins and kinases downstream able to transduce the signal, as well as plasmatic IGF1 binding proteins. IGF1 is an anabolic hormone mainly produced in the liver and also locally expressed in peripheral tissues. IGF1 is under the control of GH from the pituitary and the secretion of GH/IGF1 is essential for normal growth in children and for the maintenance of anabolic processes in adults. The activity of IGF1 is influenced by six binding proteins (IGFBPs), which also have independent biological actions [51].

Although IGF1 pathways are essential, various studies in model organisms support the hypothesis that a reduction of this signaling can exert life span-extending effects [52,53,54,55]. Considering that the levels of GH and IGF1 are down-modulated during normal or accelerated aging, whereas constitutively low IIS promotes longevity, it has been hypothesized that IIS decrease is a response to some damage naturally occurring during metabolism and cell growth. Thus, organisms with a constitutively decreased IIS have longer life span as their metabolism and damage rates are lower, while during aging the organism try to survive by decreasing IIS [56].

Low IGF1 levels in humans can predict survival in people with exceptional longevity [57] and one mechanism might be related to a lower risk of developing cancers [58]. Laron’s syndrome (LS) is a rare genetic disorder presenting a dissociation between GH and IGF1 activity. The number of known and/or published LS patients is around 350. Most cases have been reported from the Mediterranean region and Southern Ecuador, and a few cases from South America, as recently reviewed [59,60,61]. LS is characterized by insensitivity to GH. Up to the present time, over 70 mutations of GH receptor (GHR) genes have been identified leading to GH/IGF1 signaling pathway defect. The biochemical features typical of LS patients are high serum level of GH and low free IGF1 concentrations. People affected by LS are characterized by dwarfism and obesity and have a tendency to develop hyperlipidemia, but surprisingly have a very low risk to manifest aging-related pathologies such as diabetes and cancer [59,62,63]. Additionally, other studies in humans showed a positive association between low IGF1 activity and longevity [64].

However, not all studies found a correlation between low IGF1 and longevity in humans [64]. For example, in a cohort of 252 centenarians, low IGF1 and IGFBP3 serum concentrations were associated with increased mortality [65]. Another study also showed that healthy centenarians had a plasma IGF1/IGFBP3 molar ratio greater than aged subjects [66]. Moreover, in the elderly the GH/IGF1 system might elicit a protective and beneficial effect which is mainly related to its anabolic activity, especially on muscle and bone [67].

### 2.6. Mammalian Target of Rapamycin (mTOR)

The “mammalian target of rapamycin” (mTOR), is a serine/threonine protein kinase which is involved in regulating protein synthesis, cellular growth and proliferation. This enzyme receives and integrates many hormonal stimuli coming from the IIS pathway as well as signals coming from specific nutrients, in particular amino acids (AAs) like leucine [68]. mTOR can participate to two different complexes with many other proteins: mTOR complex 1 (mTORC1) and mTOR complex 2 (mTORC2). mTORC1 is implicated in most of actions exerted by this kinase. In particular, by the activation of this complex, mTOR transduces anabolic signals. Its main functions are regulation of the biogenesis of ribosomes and the synthesis of proteins and nucleotides according to cell needs. Moreover, mTOR stimulates lipogenesis, glycolysis and pentose phosphate pathway. mTOR downregulates catabolic pathways and suppresses protein turnover mainly via the inhibition of autophagy. The downregulation of mTOR activity suppresses protein synthesis and this event allows endogenous protein repair and degradation machinery to preserve the correct proteostasis and attenuating possible aggregate-related damages. The activity of mTOR is repressed in fasting or stress conditions, when decreased substrate availability counteracts anabolic pathways.

Several studies in key model organisms showed that the mTOR pathway is related to life span and health span and reduced mTOR signaling through genetic or pharmacological interventions results in life span extension in yeast, worms, flies and mice [69,70,71]. mTOR activation may exert a crucial role in aging process. Many age-associated pathologies, which are characterized by hyper-functionality of proliferative processes, may be prevented by a general decrease in protein synthesis. However, in elderly people mTOR may show the beneficial outcomes of its activity, preventing sarcopenia and lean mass loss. In conclusion, there is a general consensus that this kinase can promote metabolic health or disease depending on human age and on particular signaling of specific tissues [72].

Compared with mTORC1, much less is known about mTORC2 upstream regulation and downstream outputs, and the relationship between the two complexes is still unclear. Although the implication of mTORC2 in human health span and life span has been less studied, it is known that in mammalian cells, mTORC2 interacts with IIS-mTOR pathway activating AKT to repress FOXO1 and FOXO3, which affect longevity [73]. Moreover, the selective suppression of mTORC2 seems to reduce life span and to be associated with changes in hormone sensitivity and metabolism (for example, insulin resistance), with a negative impact on health span [74]. Thus, a possible strategy to counteract age-related pathologies and improve longevity and health span could be the use of specific inhibitors to suppress mTORC1 with minor effects on mTORC2.

## 3. Interplay Between Nutrient-Sensing Pathways

Great attention during last few decades has been focused on the study of how nutrient-sensing systems, including IIS pathway, mTOR, AMPK and SIRTs, influence life strategies such as those that determine organism physiology, health status, reproductive capacity and somatic tissue maintenance with age [75]. Indeed, a complex crosstalk exists between dietary components and nutrient-sensing pathways in most model organisms, as well as in humans. Evidence has shown that diet balance, particularly when associated to an active lifestyle, realizes its effect on life span and aging through the critical sensors of AMPK, SIRTs and mTOR and by modulating mTOR/AMPK interaction [76,77].

**Figure 1:**
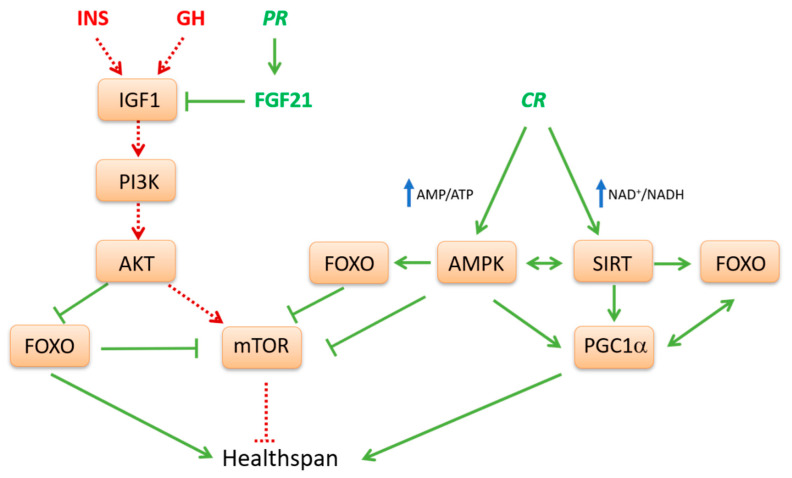
Interplay between nutrient-sensing pathways potentially affecting health span. A complex crosstalk exists between nutrient and energy-sensing pathways in mammals. Caloric restriction (CR), that is a 25–30% reduction of caloric intake without malnutrition, as well as other dietary restriction (DR) interventions such as protein restriction (PR) or low protein/carbohydrate diets represent conditions that downregulate the insulin-like growth factor 1 (IGF1) mediated pathway. A reduced signaling of this pathway leads to a decrease of AKT, which causes the inhibition of mammalian target of rapamycin (mTOR) and activation of forkhead box O transcription factor (FOXO) which, in turn, suppresses mTOR. Reciprocal modulation of mTOR and FOXO induced by CR correlates with reduced global protein synthesis and decreased cell growth and proliferation. In PR conditions, low levels of essential dietary amino acids, particularly of methionine, stimulates the expression of fibroblast growth factor 21 (FGF21), a main inhibitor of IGF1 signaling. Through these mechanisms, DR orchestrates a program of genes involved in protein homeostasis and stress resistance that positively affects health span. Under conditions of CR, increased cellular AMP/ATP and NAD^+^/NADH ratios cause activation of AMP-activated protein kinase (AMPK) and sirtuins (SIRTs), respectively. AMPK, a critical cellular energy sensor, inhibits mTOR and contributes to stimulate SIRTs, including SIRT1 and SIRT3. In turn SIRTs, through a positive loop, further favor activation of AMPK itself. Both AMPK and SIRTs activate FOXO and peroxisome proliferator-activated receptor gamma coactivator 1α (PGC1α) through phosphorylation and deacetylation, respectively. PGC1α coactivates FOXO thus favoring the expression of key antioxidant enzymes as well as pathways involved in autophagy and mitophagy. Moreover, PGC1α induces the transcription of many important genes involved in mitochondrial biogenesis, complete fatty acid oxidation and stress resistance pathways. Therefore AMPK, SIRTs, FOXO and PGC1α can engage in a positive feedback loop, thus connecting these sensors into a unified response which may improve health span. This simplified model does not account for macronutrient-specific responses or tissue-specific nutrient detection and it does not distinguish between different tissues when summarizing downstream signaling effects.

Generally, in model organisms, nutrient limitation or low protein diets are life extending via activation of AMPK-SIRT pathway (Figure 1). Conversely, nutrient abundance as well as high glucose or protein diets shorten life span and promote aging via the INS-mTOR pathway [77]. In fact, current available evidence strongly supports the idea that anabolic signaling accelerates aging and that generally catabolic signaling extends longevity [78]. In well fed conditions the IIS-mTOR pathway is activated. This pathway participates in glucose and AA sensing and its stimulation converges toward anabolism, cell growth and proliferation. Conversely, during fasting, three other interconnected nutrient-sensing systems, AMPK, SIRT1 and FOXOs are induced (Figure 1). They act in the opposite direction, drive oxidative catabolism, improve metabolic efficiency and counteract the mTOR pathway. Indeed, in most eukaryotic organisms, the pathway of GH and IGF1 and its downstream effectors, mTOR and S6K, are involved in the regulation of metabolism and growth in response to nutrient abundance and thereby promote aging [78]. Steady activation of this pathway may play a crucial role in the development of type 2 diabetes, obesity and cancer [79].

Many studies, based on humans and animal models, support the hypothesis that lower protein intake results in lower activity of the GH/IGF1 axis, thereby protecting against the development and onset of aging and age-related pathologies. Diets low in animal proteins, or low in essential AAs, stimulate the expression of FGF21, a main inhibitor of IGF1 signaling [80]. Genetic down-regulation of many IIS core-components has an evolutionarily conserved effect on longevity in model organisms [75] and elicits important beneficial effects in mammals [52]. The downregulation of the IIS pathway upstream of mTOR leads to the activation of FOXO1. FOXO1, in turn, inhibits mTOR [81,82] and activates antioxidant enzyme expression, stress response pathways and autophagy [83].

In conditions of macronutrient depletion, changes in intracellular AMP/ATP and NAD^+^/NADH ratios represent crucial signals that modulate AMPK and SIRT activities, respectively [15]. Indeed, when energy level is low, a positive loop between AMPK-SIRT1 is activated, which derepresses cellular energy generating systems and inhibits anabolic processes consuming energy, in order to achieve energetic homeostasis. In fact, these nutrient sensors and regulators counteract signals dependent on IGF1 and mTOR and, on the other hand, activate mechanisms to enhance more efficient energy production through oxidative metabolism. In this regard, the TCA intermediate α-ketoglutarate (α-KG) has been proposed as a key metabolite mediating life span extension by fasting/CR through inhibition of ATP synthase and downstream of mTOR [84]. Differently from citrate, α-KG levels increase under starvation or physical exercise, following glutamate transamination or dehydrogenation to fuel TCA cycle and provide carbons derived from AA catabolism for gluconeogenesis.

The biochemical functions of both AMPK and SIRT1 may explain many aspects related to metabolic flexibility. This means the ability to efficiently adapt metabolism depending on demand or supply [85]. In muscle response to fasting or endurance exercise, AMPK is the driving force for the switch from glucose to fatty acid oxidation. Interestingly, ineffective fat utilization in the muscle abrogates the life span effects of reduced calorie intake, thus supporting the important role of AMPK for metabolic health. Moreover, since AMPK can engage in a positive feedback loop with SIRT1, both sensors of low-energy states seem to work in concert [86]. Indeed, AMPK has been shown to promote cytosolic SIRT1 activity by increasing NAD^+^ synthesis and the NAD^+^/NADH ratio [87]. Activation of SIRT1 then leads to the deacetylation and activation of LKB1, the upstream activator of AMPK [86].

However, the relation between AMPK and SIRT1 appears even more complex, since it has been shown that the inhibition of the first can affect the expression of the latter [88]. Moreover, low calorie diets, through SIRT1, reduce the expression of inflammation mediators by counteracting NF-kB pathway. Repression of proinflammatory processes can then produce a redundant response such as autophagy activation by blocking mTOR-dependent FOXO inhibition or by inducing the activity of autophagy-related proteins (ATGs) [89]. SIRTs together with AMPK remodel transcriptional networks to control mitochondrial biogenesis and turnover via mitophagy. The critical transcriptional effector of these processes appears to be PGC1α, as it is a direct target of AMPK phosphorylation and SIRT1 deacetylation [87,90]. SIRT1 and AMPK can modulate PGC1α not only at the post-transcriptional level affecting its activity, but also at the transcriptional level promoting its expression [91,92,93].

The sum of all known activities of SIRTs on transcription factors and metabolic enzymes would program cells for oxidative metabolism in mitochondria. At the molecular level, SIRT1 might enhance oxidative metabolism and prevent hepatic lipid accumulation through the activation of PGC1α and PPARα. In particular, the colocalization with SIRT1 facilitates the efficient deacetylation of PGC1α, which can then coactivate PPARα. In the absence of SIRT1, PGC1α remains associated in a constitutively hyperacetylated state, which dampens PGC1α coactivating activity and blunts PPARα transcriptional activation [94]. Main classical targets of SIRT1 are also the transcriptional factors FOXOs. During energy starvation, both PGC1α and FOXO3 are activated through phosphorylation or deacetylation by AMPK and SIRT1, respectively [26]. Unlike phosphorylation by AKT, modification by AMPK does not appear to alter the localization of FOXO3. The role of AMPK in the regulation of FOXOs may be, first of all, to promote interaction between cofactors and FOXOs at specific target genes [30]. The transcriptional efficiency of FOXOs needs its interaction with coactivators such as PGC1α, which also plays important roles during energy depletion. In addition, FOXOs can bind and stimulate the PGC1α promoter, representing an auto-regulatory feedback mechanism [82].

In the liver, the expression of PGC1α is markedly induced upon fasting via a cyclic AMP-responsive element-binding protein (CREB)-dependent transcriptional mechanism and it is critical in maintaining gluconeogenesis to a sustainable rate for extended periods under starvation. Indeed, PGC1α promotes the expression of gluconeogenic genes by enhancing the trans-activating potential of FOXO1 [95]. Once PGC1α is activated, it also induces and coordinates gene expression regulating many metabolic and cell fate decisions, including the stimulation of mitochondrial oxidative metabolism in many tissues and fiber-type switching in skeletal muscle. PGC1α upregulates the expression of several genes of the TCA cycle and the mitochondrial fatty acid oxidation pathway. PGC1α also regulates the expression of nuclear and mitochondrial genes that encode components of the electron transport system and oxidative phosphorylation (OXPHOS) via nuclear respiratory factor (NRF) 1,2 and estrogen-related receptor α (ERRα) coactivation. These effects can increase the expression of mitochondrial transcription factor A (mtTFA), which is known to control mtDNA replication and transcription and therefore regulate cellular oxidative metabolism [96]. In fact, at a biochemical level, PGC1α activation can ensure the complete oxidation of fatty acids to CO_2_ and H_2_O through the TCA cycle and respiratory chain and prevent ectopic production and accumulation of toxic lipid metabolites inside tissues. Thus, globally, PGC1α action improves stress resistance and metabolic fitness, inhibits insulin resistance and fat accumulation and contributes to health span.

The SIRT1-AMPK-PGC1α pathway is directly implicated in the response of skeletal muscle to endurance exercise. Contraction causes alteration in AMP, Ca^2+^ and NAD^+^ levels, which leads to the activation of AMPK, calmodulin kinase and SIRT1, respectively. These signaling proteins converge on PGC1α to promote mitochondrial biogenesis, angiogenesis, changes of fiber composition and myokine secretion. Recent studies demonstrated that several myokines such as irisin and brain-derived neurotrophic factor (BDNF) are epigenetically regulated by PGC1α in skeletal muscles, thereby modulating systemic energy balance, with marked expansion of mitochondrial volume density and oxidative capacity in different target tissues [97]. This signaling pathway mediated by PGC1α represents a main molecular mechanism mediating the pleiotropic beneficial effects of exercise on metabolic homeostasis and health span [98].

## 4. Dietary Composition and Health Span

Quality of diet and the availability of key nutrients, such as glucose, fatty acids and AAs, directly influence organismal healthy status and longevity. In particular, AA and lipid composition may strongly correlate with aging and can be used as an indicator of health span. Since any change in the cell response to dietary components may be implicated in specific diseases, recently many studies are focused on nutrigenomics in order to investigate the molecular mechanisms by which macronutrients and different dietary strategies elicit their metabolic effects at cellular level.

Current available evidence indicates that the specific AA composition of the diet (protein quality) regulates metabolism, health and longevity in model organisms [99,100]. High glucose levels in medium accelerate the senescence of cultured human cells [101], while increased glucose consumption favors aging in several model organisms including yeast [102,103] and *Caenorhabditis elegans* [104,105]. This proaging effect of glucose is associated with reduced expression of SIRTs, including SIRT3. Glucose-enriched diets shorten the life span of *Caenorhabditis elegans* by downregulating AMPK and FOXO. Conversely, glucose restriction or impaired glycolysis increase intracellular NAD^+^ levels and SIRT activity, and AMPK and extend life span.

**Figure 2:**
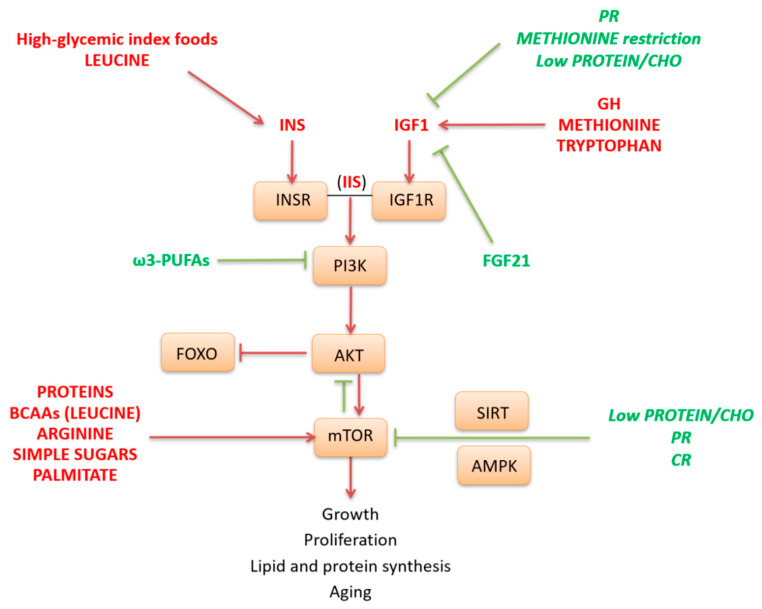
Specific nutrients and dietary manipulations affecting insulin/insulin-like growth factor signaling (IIS) pathway. IIS pathway activates the kinase mammalian target of rapamycin (mTOR) and promotes growth and aging. High-glycemic index foods as well as high intake of some amino acids (AAs) like leucine are the dietary components most involved in increasing insulin levels after feeding. Other AAs like methionine and tryptophan act on growth hormone-insulin-like growth factor 1 (GH-IGF1) pathway. Moreover, the downstream effector mTOR is directly stimulated by specific AAs including leucine and arginine, simple carbohydrates like glucose and fructose, and saturated fatty acids like palmitate. Other nutritional conditions suppress IIS pathway. In particular, this pathway is downregulated by caloric restriction (CR), protein restriction (PR) and by diets having a low ratio between proteins and carbohydrates (CHO), as well as by the stress-responsive hormone fibroblast growth factor 21 (FGF21). Other nutrients such as ω3-polyunsaturated fatty acids (ω3-PUFAs) cause mTOR repression by acting as negative modulators of fosfoinositide 3-chinasi (PI3K)/AKT pathway. Inhibition of mTOR may be also induced by CR, PR and low protein/CHO diets through mechanisms involving AMP-activated protein kinase (AMPK) and sirtuin (SIRT) activation.

The IIS pathway and its downstream target mTOR are activated in fed condition as well as by specific nutrients such as simple sugars, high glycemic index carbohydrates (CHO), and some AAs. In particular, CHO and leucine are key stimulators of insulin release, while the AAs methionine and tryptophan are key effectors of the GH-IGF1 pathway. Globally, mTOR activation requires a permissive hormonal state for anabolism, as signaled by the IIS pathway. In fact, the IIS, through AKT, leads to the activation of mTOR and inhibition of FOXO (Figure 2). Activated mTOR, in turn, inhibits AKT and can negatively regulate IIS through inhibition of insulin receptor substrate 1 (IRS1). This negative feedback loop between mTOR and insulin signaling has been causally linked to a condition of insulin resistance. The kinase mTOR is a central regulator of cell growth and proliferation in response to growth factors and nutrients. It is involved in anabolic processes such as protein, lipid and nucleotide synthesis and may play a crucial role in aging. mTOR pathway may be directly activated by different nutrients such as animal proteins, specific AAs as leucine and arginine [106,107,108], simple sugars as glucose or fructose [109], as well as by the saturated fatty acid palmitate. Conversely, mTOR is repressed by low protein and calorie diets and by ω-3 polyunsaturated fatty acids (PUFAs), which also act as negative modulator of PI3K-AKT pathway [110] (Figure 2).

Low levels of animal proteins or methionine or diets with a low protein/CHO ratio are all conditions that are able to decrease the IGF1 pathway, thus reducing mTOR stimulation [38,80,111]. Moreover, IGF1 may be blunted by scarcity of essential AAs through a pathway involving increased transcription of FGF21. This endocrine messenger selectively inhibits IGF1 signaling to conserve energy and to downregulate the GH effects on somatic growth [36,37,112]. In liver FGF21 also counteracts mTOR pathway. Moreover, FGF21 is able to exert systemic effects and different metabolic responses in other tissues.

### 4.1. Caloric Restriction (CR)

To date, evidence has shown that a reduced caloric intake, without malnutrition, represents the most robust intervention to increase life span in model organisms, including primates, and to delay the emergence of age-related diseases [113,114,115]. CR is defined as a dietary manipulation consisting in decrease total caloric intake of about 25–30% without lack of essential nutrients. Throughout the literature, CR is often used interchangeably with dietary restriction (DR). However, more exactly, CR is a partial example of DR. DR protocols include restriction of energy (CR), reduction of specific macronutrients or change in the ratio of them as well as other different feeding interventions, such as intermittent fasting or alternate day feeding [116,117].

CR produces broad effects and may improve multiple metabolic pathways, generating benefits to the whole organism. Among the main positive effects of CR, a decrease in oxidative damage and induction of autophagy are two of the hallmarks [89]. Common features of CR-dependent mechanisms include energy-saving processes, such as the maintenance of mitochondrial function in order to ensure the integrity of existing cellular components, increasing oxidant scavenging, and the induction of autophagy to recycle macromolecules and damaged organelles [118,119,120]. CR pleiotropic effects are considered as crucial strategies, well conserved throughout the course of evolution, in order to save fuel and reduce those processes and pathways not essential for survival. The main aim is to engage in activities that reuse and recycle resources and utilize them efficiently later in life, thus maximizing long-term survival. In this condition, protein synthesis is maintained to preserve somatic tissue homeostasis [121]. The mechanisms underlying the beneficial effects of CR are mainly linked to inhibition of anabolic processes, improved mitochondrial energy metabolism and switch in substrate utilization. Biochemically this is translated in a decreased reliance on glucose and its metabolites for energy and higher fatty acid oxidation. This strategy is strictly related to reduced mTOR signaling, decreased IIS pathway, as well as activation of SIRT-AMPK-PGC1α pathway. In mammals, CR may restore decreased levels of PGC1α induced by aging [15,89].

However, much longer and larger studies should clarify the effect of CR on human health. In any case, in order to prevent one side effect that might be associated with this regime such as reduced bone mineral density and sarcopenia, regular exercise results to be recommended. For these reasons, such dietary intervention in elderly people should be made with caution.

### 4.2. Caloric Restriction Mimetic (CRM) Compounds

During the last two decades, several small molecules and dietary manipulations have been developed that have been found to delay the onset of age-related diseases and increase health span and life span in different animal models, including nonhuman primates [2,78,122]. Many of these compounds have been generally named as CRMs that extend life span through an improvement of metabolic function, especially through mitochondrial metabolic reprogramming. CRMs may act at different levels, that are either targeting gut nutrient utilization and energy metabolism systems (upstream-type CRMs) or affecting downstream the major intracellular signaling pathways and nutrient-sensing pathways (downstream CRMs) as discussed above, whose dysregulation contributes to the emergence of aging phenotypes and disease [123]. Some upstream CRMs interfere with nutrient absorption/digestion, such as orlistat, an inhibitor of pancreatic lipases; acarbose, an α-glycosidase and α-amylase inhibitor, and chitosan, which reduces absorption of lipids and carbohydrates and has glucose- and fat-lowering effects. Examples of α-amylase and α-glucosidase inhibitors also include cinnamon, curcumin and phytochemicals of sweet potatoes consumed by Okinawa people. Other upstream CRMs act as glycolytic inhibitors, as 2-deoxy-D-glucose and D-glucosamine, component of chitin, which also induces fatty acid metabolism and mitochondrial respiration via AMPK. D-glucosamine has been shown to prolong life span in model organisms [124,125].

Recently, downstream CRM have been better studied. These compounds are able to affect nutrient sensors and nutrient-sensing pathways and autophagy, and may lead to epigenetic changes. They include:

(1) The classical mTOR inhibitor, rapamycin and novel drugs as rapalogs, having the same molecular scaffold as rapamycin, but with different physiochemical properties [126].
(2) Drugs targeting the NAD^+^-dependent SIRTs, such as the polyphenol resveratrol, activators of SIRTs and AMPK; other plant-derived metabolites or synthetic drugs having better selectivity for SIRTs named SIRT-activating compounds (STACs). Natural STACs include flavones, chalcones and anthocyanidins [127].
(3) Drugs targeting NAD^+^ biosynthesis, as nicotinamide mononucleotide (NMN) and nicotinamide riboside (NR), recently tested in humans [128].
(4) Drugs targeting insulin signaling pathways, CHO and fat metabolism, as the biguanide class of antidiabetic drug metformin, known activator of AMPK. Activators of AMPK are also most of the above cited phytonutrients, including resveratrol, green tea polyphenols, quercetin, etc.
(5) Drugs targeting autophagy and inducing epigenetic changes, such as spermidine. This compound is a ubiquitous natural polyamine involved in a wide range of cellular processes thanks to the endogenous synthesis which occurs in all living species. Spermidine has been shown to extend life span in several animal species [129]. In humans, spermidine supplementation resulted to be safe and to have positive effects on cognitive function of older adults [130]. Polyamines, and in particular spermidine, can be found both in animal and vegetable foods such as wheat, mushrooms, fish and cheese and such a dietary component could be particularly useful during aging, when de novo synthesis tends to decrease. Human epidemiological studies correlated dietary polyamine intake with reduced cardiovascular and cancer-related mortality [131]. Moreover, emerging evidence suggests that spermidine may exert a protective effect against an age-related pathology, such as osteoarthritis [132]. Madeo and Eisenberg described the role of this polyamine in aging and diseases [133]. More recently the health benefits of CRM compounds and nutraceuticals and their specific targets have been extensively summarized in a review [134].

### 4.3. Protein Requirement

New evidence has revealed that dietary composition and the balance of macronutrients, rather than total energy intake, may play a more important role in health span and life span extension than previously attributed [119,135]. Therefore, altering the macronutrient composition of the diet while keeping the total number of calories constant is an intriguing alternative to CR and may be even more sustainable [33]. Several short-term clinical trials have demonstrated beneficial health effects of high-protein/low-CHO diets, largely dependent on weight loss, enhanced postprandial satiety and energy expenditure [136]. However, these studies do not take into account the long-term impact of these diets. Recent studies have shown that among the different macronutrients, proteins may have a great impact on longevity and metabolic health [80]. Moreover, the amount and source of dietary proteins may differently affect aging and human health [99,100]. Higher intake of animal proteins, especially from red and processed meat consumption have been associated with increased premature death, possibly owing to systemic oxidative stress, inflammation, heme iron and endogenous formation of N-nitroso compounds [137,138]. In accordance, high animal-based protein consumption having higher tryptophan, methionine and leucine levels, is associated with higher all-cause mortality, cardiovascular and cancer mortality, whereas vegetable-based protein intake is associated with lower all-cause mortality and cardiovascular mortality, thus suggesting a great importance of protein source [139]. In particular, consumption of dietary plant proteins in adult age has been related to reduced cardiovascular risk factors, including lower systolic and diastolic blood pressure, improved lipid and lipoprotein profiles, decreased concentrations of circulating IGF1 and decreased overall mortality and cause-specific mortality [140]. Preliminary evidence has shown that in adult humans in the absence of CR, even short-term low protein diets can reduce fat mass, optimize blood glucose, decrease IGF1 levels and limit tumor onset [141]. These nutritional strategies improve metabolic health, reduce blood glucose and IGF1 levels, promote lean physical appearance and decrease the risk of developing diabetes [78]. Some studies have suggested that protein restriction (PR), restriction of specific AAs, or a vegetable protein-based diet elicit the same positive effects of CR on health status and age-related diseases [121,142,143].

The effects of low methionine intake have been studied best. It has been shown that methionine restriction improves glucose homeostasis and exerts a beneficial effect on lipid metabolism by promoting the activation and the oxidation of fatty acids in liver, activating lipolysis in WAT and increasing UCP1 expression in BAT [144,145,146,147]. As previously described, FGF21 represents a critical mediator of the metabolic effects of protein- or methionine-restricted diets [148]. In animal models, methionine restricted diets mimic both DR and PR with regard to phenotypes associated with life-span extension and induce similar gene expression and physiological changes [143]. In particular, methionine restriction decreased mitochondrial reactive oxygen species, serum glucose, insulin and IGF1 levels and altered lipogenic/lipolytic balance contributing to loss of adiposity and to health span [149].

The restriction of other AAs such as tryptophan or leucine has also been investigated. In particular, the reduced intake of tryptophan delays tumor incidence and one recent study showed clear modulation of gut hormones, weight loss, energy balance and gut microbiota in rats subjected to tryptophan restriction [150]. Moreover, restriction of leucine produces decreases in genes associated with lipogenesis through reduced expression of SREBP1c and improves insulin sensitivity. Modulation of these parameters is causally linked to weight loss, to a reduction of fat mass and to an improvement of health span. Activation of AMPK, inhibition of insulin and IGF1 pathways as well as a decrease in mTOR and in its downstream S6 kinase 1 signaling seem to be involved [151,152]. However, it is imperative to perform in humans more nutritional intervention studies with AA restriction.

One of the most difficult topics regarding the association between nutrition and longevity is the optimal extent/quantity of (age-dependent) human protein intake. On the one hand, epidemiological studies suggest that protein-rich diets, especially from animal sources, are associated with a higher risk of diseases, on the other hand, higher protein intakes result to be particularly important for elderly people in order to increase muscle mass and strength [153], which are both independent predictors of mortality. In fact, recent studies showed that in humans protein requirement and the response to AA intake levels are highly dependent on age, as well as on physiological states such as pregnancy and breastfeeding. Studies of Longo’s team concluded that humans under 65 consuming high-proteins, that is, at least 20% of their calories, were at much higher risk of illness and death from cancer than those who had 10% or fewer [154]. Moreover, clinical studies that compared the impacts of protein sources in humans under 65 have suggested that high levels of animal proteins may have negative effects on metabolic health, while vegetable proteins do not show such adverse roles [155]. However, in humans, a low protein intake appeared to benefit for groups of 50–65 years of age (adult age) but can be detrimental when applied to older ages [149], when the levels of IGF1 are naturally low. In fact, after 65 years of age, a low protein diet results in an opposite effect on all-cause and cancer-related mortality, while a higher protein intake is required to protect from sarcopenia and frailty [80,143,148] and to increase muscle mass and strength [153]. However, quite recently a vast meta-analysis reviewing 32 studies has been published that correlated protein intake and reported risk estimates for all-cause, cardiovascular and cancer mortality in adults aged 19 or older [156]. The authors concluded that a high intake of total proteins was associated with a lower risk of all-cause mortality compared with low intake. Intake of plant proteins was associated with an 8% lower risk of all-cause mortality and a 12% lower risk of cardiovascular disease mortality, while intake of animal proteins was not significantly associated with risk of cardiovascular disease and cancer mortality.

## 5. Nutrients as Epigenetic Modulators of Health Span and Longevity

Although studies on the beneficial mechanisms of nutrients and dietary habits have focused on evolutionarily conserved pathways that regulate energy metabolism and growth (for example, IIS and mTOR pathway), a large body of research indicates that nutritional and metabolic factors could influence health span and longevity also by affecting the epigenetic program. In fact, even if the genetic inheritance is crucial to determine the expression of a feature or a trait in an organism, actually differential parent-specific epigenetic modifications can occur during gametogenesis by establishing the expression of one gene copy rather the other one. Twin studies suggested that genetics at birth determines only 25% of life span, therefore, epigenetic factors have a crucial role in affecting both human health span and aging. Thus, epigenetic regulation has been identified as a key mechanism to regulate age-related phenotype and age-related diseases [157]. The gene expression profile can be relatively easily modified by the environmental impact related to the lifestyle, including dietary changes, physical exercise, pharmaceuticals and even gut microbiota. In particular, the intake of specific nutrients or nutraceuticals with antioxidant and anti-inflammatory effects may improve health status by affecting transcription and translation and gene expression profile [1]. This is an emerging and particularly interesting field of research. Moreover, compounds from the diet do not only act through direct regulation of epigenetic marks, but evidence is growing that these effects might be also indirect, through modification of the physiology of the gut microbiota [158,159].

The epigenetic mechanisms include DNA methylation, histone post-translational modifications (PTM) and post-transcriptional regulation of gene expression by noncoding RNAs (microRNAs, long noncoding RNAs). These processes are able to regulate synergistically and cooperatively gene expression by changing chromatin organization, DNA accessibility, mRNA degradation and protein translation [158].

DNA methylation is widely regarded as the most stable epigenetic mark and is usually associated with transcriptional gene repression. The most abundant type of DNA methylation in eukaryotes is cytosine 5-methylation, mainly at the level of CpG islands (genomic regions with high CG:GC ratio), but also at other dinucleotides, such as CpA, CpT, and CpC. These modifications are mediated by DNA methyltransferases (DNMTs) which transfer a methyl group from S-adenosylmethionine (SAM) to the acceptor nucleotide. The epigenetic clock theory of aging has been proposed by identifying DNA methylation signatures able to predict biological age and disease susceptibility [160].

Nutritional and dietary factors have been postulated to affect DNA methylation by changing the availability of the methyl donors and altering the activity of the DNMT enzymes [161]. Folate and related B-vitamins as well as key nutrients in 1-carbon metabolism, the main metabolic pathway for providing methyl groups for DNA methylation, are essential factors that can modify epigenetic age. Aberrant pattern of DNA methylation, due to B-group vitamin deficiency or to polymorphisms affecting different enzymes involved in this metabolism, could influence epigenetic and thus chronological age. Supplementation with B-vitamins, predominantly folate and vitamin B-12, or dietary intake of these nutrients can modify DNA methylation program in elderly subjects as reported in an epigenome-wide association study (EWAS) [162], proposing these biofactors as crucial targets to further investigate in the field of nutrigenomics. Another EWAS has highlighted that low dietary intake of riboflavin was associated with increased level of methylated CpG at a specific genomic locus (cg21230392 in the first exon of PROM1) [163]. Selenium supplementation reduces methylation potential, DNA methyltransferase activity and DNA methylation by reducing homocysteine concentration and causing an imbalance in the methionine-homocysteine cycle [164]. Flavanols and polyphenols too may influence the activity of enzymes including DNMTs and thus impact methylation processes. Selenium, genistein, quercetin, curcumin and green tea polyphenols are able to modulate DNMT activity. Epigallocatechin-gallate (EGCG), a key polyphenol in tea, inhibits DNMTs directly by fitting to the binding pocket and it leads to demethylation and reactivation of methylation-silenced genes in cancer cells. Indeed, many flavonoids from tea and fruits are able to reverse hypermethylation and, in this manner, to reactivate tumor suppressor genes [165].

Gene expression may be also regulated by changes of chromatin structure by post-translational modifications (PTMs) of histones. These DNA-binding proteins can undergo different kind of modifications (methylation, phosphorylation, acetylation, ubiquitylation, and sumoylation) and in different regions (nucleosomes and/or N-terminal tails). Depending on the type of modification, the impact on chromatin accessibility and stability differs considerably. Histone methylation mostly leads to a more condensed chromatin structure and repress gene expression, acetylation plays an opposing role by relaxing it and promoting gene expression. These modifications are finely mediated by histone acetyltransferases (HATs), which transfer an acetyl group from acetyl-CoA on specific lysine residues in the histone tail and histone methyltransferases (HMTs) that can mono-, di- and tri-methylate lysine residues. Furthermore, histone deacetylases (HDACs and SIRTs) and histone demethylases (HDMs), counteract the action of HATs and HMTs contributing to the chromatin condensation and gene expression [166]. Among SIRTs, SIRT1 is responsible of the direct deacetylation of lysine residues of different histones. Moreover, this enzyme may influence the level and activity of other enzymes involved in histone modifications. All these interactions usually keep the chromatin in a closed state resulting in gene silencing. SIRT1 can also modulate the activity of the p300 HAT [167,168]. SIRT6 is mainly a histone H3 lysine 9 (H3K9) deacetylase. By this action it can prevent telomeric shortening. Telomeres are dynamic DNA structures at the chromosome end that consist in tandem repeats of nucleotide (TTAGGG) associated to proteins. They are lost in somatic cells after each replication process and this can be prevented in germ, stem and many cancer cells thanks to the activity of specialized proteins, the reverse transcriptase telomerase. These enzymes add telomeric sequence to the ends of chromosomes, saving the telomere length. This shortening process is associated with cellular senescence when telomeres are too short to replicate and divide, and thus with cellular aging [169]. Telomere length (TL) is critical for cell proliferation and shortens with age. An accelerated shorting has been also related to an increased intake of red and processed meat, sweetened carbonated beverages, sodium and white bread. On the other hand, a significant positive association between the consumption of a Mediterranean diet and TL was shown [170]. Oxidative stress has emerged as a critical inducer of telomere shortening and thus, antioxidant supplementation and/or a healthy diet rich in natural free radical scavengers can prevent this process and potentially slow cellular aging [171].

The post-transcriptional regulation of gene expression is an additional epigenetic mechanism exerted by non-coding RNAs. Within this family, the most studied members are miRNAs. These molecules are small (21–25 nucleotides) RNAs, able to repress the expression of specific complementary mRNAs that are recognized thanks to the base-pairing between the miRNA “seed sequence” and the mRNA complementary one. Although the direct involvement of some miRNAs in the regulation of metabolism has been highlighted in many studies, the specific role as beneficial or detrimental modulators should be considered as complex and highly context-dependent. In this regard, in a recent paper, the role of miRNA-128-1 has been investigated, located at the center of a positively selected locus responsible of a thrifty phenotype [172]. In particular, an altered expression of this miRNA could have been beneficial in ancient times as adaptation to famine, but at the present, with overnutrition, it could predispose to obesity and type 2 diabetes.

Slattery and colleagues have demonstrated that diet and lifestyle factors regulate miRNA level in carcinoma and normal colorectal tissues. They assessed 34 diet and lifestyle variables with miRNA expression levels. These variables that are able to alter miRNA expression fell into two categories: (1) dietary carbohydrate, sucrose and whole grains, and (2) NSAIDs (nonsteroidal anti-inflammatory drugs) and oxidative balance score, that are implicated in inflammation and oxidative stress [173]. Other associations have been observed between folate consumption and let-7a, miRNA-21, miRNA-23, miRNA-130, miRNA-190, miRNA-17-92, and miRNA-122 in liver samples, between retinoic acid and let-7a, miRNA-15a/miRNA-16-1, and miRNA-23 in acute promyelocytic leukemia [174], resveratrol and miRNA-663, miRNA-155, miRNA-21, miRNA-181b and miRNA-30c2 in breast tissue cells [175]. These studies support the hypothesis that miRNAs are regulated by diet and lifestyle factors and, in turn, they can mediate cellular response to different diet and habits adopted. Emerging evidence in recent years indicates that miRNAs included in foods, such as plants or milks, can be absorbed into the blood and reach the different organs where they can modulate gene expression and thus cellular pathways. Despite the literature on this fascinating topic, many researchers have their doubts considering the possibility that the “diet-derived” miRNAs were actually the result of contamination [176].

## 6. Specific Dietary Pattern and Health Span

### Dietary Models

The existing data clearly showed that dietary habits, i.e., what we eat and how much we consume, have relevant roles in determining our health span. Several studies showed that a high intake of (red) meat and especially processed meat, as well as animal proteins, simple sugar, high-glycemic index carbohydrates and the saturated fatty acid palmitate having a higher inflammatory potential were positively associated with all-cause mortality. In addition, the Mediterranean diet (MD) as well as high-quality diets, containing whole grains, vegetables, fruits, nuts, ω-3 PUFAs and also coffee and green tea was associated with a reduced all-cause mortality risk [177]. At this purpose, the nutritional and lifestyle behavior of people having a longer life span than average, such as those living in Sardinia (Italy), Okinawa (Japan) and Loma Linda (California, USA), has been intensively studied [178,179]. Factors which are associated with longevity in these populations are regular physical activity, social engagement and purposeful living, spirituality, maintenance of normal body mass and eating in moderation a healthy diet with high consumptions of vegetables, fruits, whole grains and ω-3 PUFAs [178,180].

Okinawans’ have an active lifestyle and consumed approximately 17% fewer calories than the average adult in Japan and a 10–15% deficit in energy intake when estimated according to the Harris–Benedict equation of the energy requirements [181]. The typical Okinawan diet (OD) is low in protein (9% of calories), mainly from soy and fish and rich in fresh vegetables. These foods are high in ω-3 PUFAs and phenolic compounds. Besides the traditional OD, for several decades, the MD has also been associated with various health aspects related to healthy aging and longer life span [182,183]. Both MD and OD provide minerals, folic acid, vitamins E, C, ω-3 PUFAs, fibers and phytonutrients (polyphenols, phytosterols, carotenoids, terpenoids, etc.) [184]. In particular, MD supplies whole grain cereals, nuts, pulses, wine and olive oil. Although MD contains more proteins, these come mainly from fish and legumes, which represent a good protein source. MD also includes olive oil, a great source of the monounsaturated oleic acid as well as of healthy phytonutrients, for example hydroxytyrosol. This polyphenol has been reported able to exert beneficial effects against metabolic syndrome, hypertension, cardiovascular [185], neurodegenerative diseases [186] and age-related diseases like osteoarthritis [187,188]. Different studies have shown that MD may reduce mortality from cancer [189], exert anti-inflammatory effects [190] and decrease oxidative stress markers [191]. With respect to MD, OD includes more fresh vegetables and fruits. It also includes curcumin-rich foods best known for antioxidant properties, as well as high levels of sweet potatoes, which are rich of fibers, vitamins and anthocyanins having multiple health benefits.

Typically, both MD and OD have low energy density, but are high in nutritional diversity and are sources of polyphenols, belonging to compounds named “nutraceuticals”. These phytonutrients well considered as “drugs for healthy people” can exert preventive and protective functions for human health. They have been also referred as “hormetins”, since they may act in a hermetic manner, stimulating several stress-response pathways including the AMPK-SIRT-FOXO-PGC1α network that finally would lead to health benefits [192]. Favorable effects of these dietary models have been also in part related to the low content of saturated fatty acids, full-fat dairy products, animal proteins, refined grains and simple sugars, which might down-modulate the insulin/IGF1 or the mTOR pathways [182].

## 7. Conclusions

Due to the prevalence of malnutrition (particularly overnutrition) along with obesity rises worldwide, as well as pathologies favored by wrong dietary habits, the interest of the scientific community has shifted toward expansion of nutritional sciences and nutrigenomics in the hope to add health and years to people’s life. Taking into account that dietary regimes can largely influence overall human health, new findings are of great importance to fully realize the interplay between dietary components and human health. In particular, the molecular mechanisms by which individual nutrients, the balance of them or some dietary conditions may interact with different genes and pathways are currently being uncovered. There is mounting evidence that the manipulation of dietary components or restriction of total energy may be often associated with better health outcomes in humans [155,193,194,195,196,197]. Therefore, altering the macronutrient composition of the diet while keeping the total number of calories constant is an intriguing alternative that may be more sustainable [33] and needs further investigation. Actually, among the different types of macronutrient restriction, reduced intake of proteins and AAs represents the most effective prolongevity intervention in certain conditions [114,149]. However, the positive effects of specific AA restriction on metabolic health in humans warrant further investigation.

Since the interaction between diet and nutrient-sensing pathways may play a vital role in affecting organism physiology and health, the major aim across future lines of research will be to fully address the mechanistic insights of the role by which nutritional components and nutraceuticals may have an impact on nutrient sensors and pathways in order to promote health span and prevent pathological conditions. Genome-wide-associated studies from human and animal models and the ongoing research of molecular mechanisms underlying diseases point out the potential benefits of specific, maybe personalized, dietary interventions as novel interesting antiaging strategies to reduce the development and the deleterious impact of many age-related diseases and improve health and health span.

Actually, although DR has been reported to provide benefits on health and extend life span in almost all model organisms tested, several studies suggest that the DR response is not universal, and that genetics strongly affect the potential results [198,199]. Indeed, there is increasing evidence of variation in the response to DR both within and between populations and genotypes, with results dramatically ranging from life span extension to non-significant response or even reduced life span [200]. This heterogeneity in response to treatment is expected to be equally—if not more—present in human populations and represents an issue that needs to be addressed for translation to preventive and personalized medicine [199].

A better identification of the effectors of nutrients and bioactive compounds might represent a key issue in future research in the nutritional biochemical field. Now it is known that the activation of AMPK and/or deacetylases and the inhibition of mTOR pathways, IIS pathway and/or acetylases represent crucial targets. Moreover, further research is needed to clarify in humans the complex network of interactions and protein modifications underlying the metabolic implications of different dietary models that ensure higher energetic efficiency and beneficial clinical outcomes. Presently, it is clear that modulation of SIRT1 activity is, together with AMPK activity and PGC1α-dependent pathways, at the center of a protective response. These pathways could mediate, among other things, the response to classical antiaging effectors such as CR, CRMs, exercise or “exercise mimetics”. Nutritional strategies able to reduce the development of degenerative diseases, counteract processes related to aging (such as organelle dysfunctions, inflammation, oxidative stress, DNA instability, sarcopenia, etc.) as well as reactivate functions that are repressed with aging (autophagy, proteostasis) will be more and more topical in the future.

Moreover, in our opinion, future studies will be also needed to understand the interindividual differences in the response to different dietary models, depending on genetic background, gut microbiota shaping, sex, physical activity or mitochondrial capacity as well as to assess the long-term implications in relation to health span or prevention of age-related diseases of the most personalized dietary interventions. The challenge of the next century is to test whether direct intervention on the aging process can result in a similar beneficial effect on both life span and health span.

## References

[1] Ekmekcioglu C. (2019). Nutrition and longevity—From mechanisms to uncertainties. Crit. Rev. Food Sci. Nutr..

[2] Heiss C., Spyridopoulos I., Haendeler J. (2018). Interventions to slow cardiovascular aging: Dietary restriction, drugs and novel molecules. Exp. Gerontol..

[3] Sen P., Shah P.P., Nativio R., Berger S.L. (2016). Epigenetic Mechanisms of Longevity and Aging. Cell.

[4] Longo V.D. (2019). Programmed longevity, youthspan, and juventology. Aging Cell.

[5] Li X., Kazgan N. (2011). Mammalian sirtuins and energy metabolism. Int. J. Biol. Sci..

[6] Pande S., Kratasyuk V.A., Medvedeva N.N., Kolenchukova O.A., Salmina A.B. (2017). Nutritional biomarkers: Current view and future perspectives. Crit. Rev. Food Sci. Nutr..

[7] Zhao S., Xu W., Jiang W., Yu W., Lin Y., Zhang T., Yao J., Zhou L., Zeng Y., Li H. (2010). Regulation of Cellular Metabolism by Protein Lysine Acetylation. Science.

[8] Ponugoti B., Kim D.-H., Xiao Z., Smith Z., Miao J., Zang M., Wu S.-Y., Chiang C.-M., Veenstra T.D., Kemper J.K. (2010). SIRT1 Deacetylates and Inhibits SREBP-1C Activity in Regulation of Hepatic Lipid Metabolism. J. Biol. Chem..

[9] Picard F., Kurtev M., Chung N., Topark-Ngarm A., Senawong T., Machado de Oliveira R., Leid M., McBurney M.W., Guarente L. (2004). Sirt1 promotes fat mobilization in white adipocytes by repressing PPAR-γ. Nature.

[10] Baur J.A., Ungvari Z., Minor R.K., Le Couteur D.G., de Cabo R. (2012). Are sirtuins viable targets for improving healthspan and lifespan?. Nat. Rev. Drug Discov..

[11] Guarente L. (2013). Calorie restriction and sirtuins revisited. Genes Dev..

[12] Bai X., Yao L., Ma X., Xu X. (2018). Small Molecules as SIRT Modulators. Mini-Rev. Med. Chem..

[13] Escobar K.A., Cole N.H., Mermier C.M., VanDusseldorp T.A. (2019). Autophagy and aging: Maintaining the proteome through exercise and caloric restriction. Aging Cell.

[14] Ng F., Tang B.L. (2013). Sirtuins’ modulation of autophagy. J. Cell. Physiol..

[15] Ruetenik A., Barrientos A. (2015). Dietary restriction, mitochondrial function and aging: From yeast to humans. Biochim. Biophys. Acta.

[16] Qiu X., Brown K., Hirschey M.D., Verdin E., Chen D. (2010). Calorie Restriction Reduces Oxidative Stress by SIRT3-Mediated SOD2 Activation. Cell Metab..

[17] Sundaresan N.R., Gupta M., Kim G., Rajamohan S.B., Isbatan A., Gupta M.P. (2009). Sirt3 blocks the cardiac hypertrophic response by augmenting Foxo3a-dependent antioxidant defense mechanisms in mice. J. Clin. Investig..

[18] Donniacuo M., Urbanek K., Nebbioso A., Sodano L., Gallo L., Altucci L., Rinaldi B. (2019). Cardioprotective effect of a moderate and prolonged exercise training involves sirtuin pathway. Life Sci..

[19] Barja G. (2019). Towards a unified mechanistic theory of aging. Exp. Gerontol..

[20] Bai P., Canto C., Oudart H., Brunyanszki A., Cen Y., Thomas C., Yamamoto H., Huber A., Kiss B., Houtkooper R.H. (2011). PARP-1 inhibition increases mitochondrial metabolism through SIRT1 activation. Cell Metab..

[21] Xu C., Wang L., Fozouni P., Evjen G., Chandra V., Jiang J., Lu C., Nicastri M., Bretz C., Winkler J.D. (2020). SIRT1 is downregulated by autophagy in senescence and ageing. Nat. Cell Biol..

[22] Hardie D.G., Schaffer B.E., Brunet A. (2016). AMPK: An Energy-Sensing Pathway with Multiple Inputs and Outputs. Trends Cell Biol..

[23] Hardie D.G., Ross F.A., Hawley S.A. (2012). AMPK: A nutrient and energy sensor that maintains energy homeostasis. Nat. Rev. Mol. Cell Biol..

[24] Garcia D., Shaw R.J. (2017). AMPK: Mechanisms of Cellular Energy Sensing and Restoration of Metabolic Balance. Mol. Cell.

[25] Viollet B., Guigas B., Leclerc J., Hébrard S., Lantier L., Mounier R., Andreelli F., Foretz M. (2009). AMP-activated protein kinase in the regulation of hepatic energy metabolism: From physiology to therapeutic perspectives. Acta Physiol..

[26] Greer E.L., Oskoui P.R., Banko M.R., Maniar J.M., Gygi M.P., Gygi S.P., Brunet A. (2007). The energy sensor AMP-activated protein kinase directly regulates the mammalian FOXO3 transcription factor. J. Biol. Chem..

[27] Burkewitz K., Zhang Y., Mair W.B. (2014). AMPK at the Nexus of Energetics and Aging. Cell Metab..

[28] Day E.A., Ford R.J., Steinberg G.R. (2017). AMPK as a Therapeutic Target for Treating Metabolic Diseases. Trends Endocrinol. Metab..

[29] Piskovatska V., Stefanyshyn N., Storey K.B., Vaiserman A.M., Lushchak O. (2018). Metformin as a geroprotector: Experimental and clinical evidence. Biogerontology.

[30] Brown A.K., Webb A.E. (2018). Regulation of FOXO Factors in Mammalian Cells. Curr. Top. Dev. Biol..

[31] Murtaza G., Khan A.K., Rashid R., Muneer S., Hasan S.M.F., Chen J. (2017). FOXO Transcriptional Factors and Long-Term Living. Oxid. Med. Cell Longev..

[32] Link W. (2019). Introduction to FOXO Biology. Methods Mol. Biol..

[33] Fontana L., Partridge L. (2015). Promoting health and longevity through diet: From model organisms to humans. Cell.

[34] Mercken E.M., Crosby S.D., Lamming D.W., JeBailey L., Krzysik-Walker S., Villareal D.T., Capri M., Franceschi C., Zhang Y., Becker K. (2013). Calorie restriction in humans inhibits the PI3K/AKT pathway and induces a younger transcription profile. Aging Cell.

[35] Urbánek P., Klotz L.O. (2017). Posttranscriptional regulation ofFOXOexpression: MicroRNAs and beyond. Br. J. Pharmacol..

[36] Salminen A., Kaarniranta K., Kauppinen A. (2017). Regulation of longevity by FGF21: Interaction between energy metabolism and stress responses. Ageing Res. Rev..

[37] Zhang Y., Xie Y., Berglund E.D., Coate K.C., He T.T., Katafuchi T., Xiao G., Potthoff M.J., Wei W., Wan Y. (2012). The starvation hormone, fibroblast growth factor-21, extends lifespan in mice. Elife.

[38] Lees E.K., Król E., Grant L., Shearer K., Wyse C., Moncur E., Bykowska A.S., Mody N., Gettys T.W., Delibegovic M. (2014). Methionine restriction restores a younger metabolic phenotype in adult mice with alterations in fibroblast growth factor 21. Aging Cell.

[39] Hill C.M., Berthoud H.-R., Münzberg H., Morrison C.D. (2018). Homeostatic sensing of dietary protein restriction: A case for FGF21. Front. Neuroendocrinol..

[40] Solon-Biet S.M., Cogger V.C., Pulpitel T., Heblinski M., Wahl D., McMahon A.C., Warren A., Durrant-Whyte J., Walters K.A., Krycer J.R. (2016). Defining the Nutritional and Metabolic Context of FGF21 Using the Geometric Framework. Cell Metab..

[41] BonDurant L.D., Potthoff M.J. (2018). Fibroblast Growth Factor 21: A Versatile Regulator of Metabolic Homeostasis. Annu. Rev. Nutr..

[42] Sonoda J., Chen M.Z., Baruch A. (2017). FGF21-receptor agonists: An emerging therapeutic class for obesity-related diseases. Horm. Mol. Biol. Clin. Investig..

[43] Perez-Marti A., Garcia-Guasch M., Tresserra-Rimbau A., Carrilho-Do-Rosario A., Estruch R., Salas-Salvado J., Martinez-Gonzalez M.A., Lamuela-Raventos R., Marrero P.F., Haro D. (2017). A low-protein diet induces body weight loss and browning of subcutaneous white adipose tissue through enhanced expression of hepatic fibroblast growth factor 21 (FGF21). Mol. Nutr. Food Res..

[44] Vainshtein A., Sandri M. (2020). Signaling Pathways That Control Muscle Mass. Int. J. Mol. Sci..

[45] Tezze C., Romanello V., Sandri M. (2019). FGF21 as Modulator of Metabolism in Health and Disease. Front. Physiol..

[46] Oost L.J., Kustermann M., Armani A., Blaauw B., Romanello V. (2019). Fibroblast growth factor 21 controls mitophagy and muscle mass. J. Cachexia Sarcopenia Muscle.

[47] Hanks L.J., Gutiérrez O.M., Bamman M.M., Ashraf A., McCormick K.L., Casazza K. (2015). Circulating levels of fibroblast growth factor-21 increase with age independently of body composition indices among healthy individuals. J. Clin. Transl. Endocrinol..

[48] Tezze C., Romanello V., Desbats M.A., Fadini G.P., Albiero M., Favaro G., Ciciliot S., Soriano M.E., Morbidoni V., Cerqua C. (2017). Age-Associated Loss of OPA1 in Muscle Impacts Muscle Mass, Metabolic Homeostasis, Systemic Inflammation, and Epithelial Senescence. Cell Metab..

[49] Ji K., Zheng J., Lv J., Xu J., Ji X., Luo Y.-B., Li W., Zhao Y., Yan C. (2015). Skeletal muscle increases FGF21 expression in mitochondrial disorders to compensate for energy metabolic insufficiency by activating the mTOR–YY1–PGC1α pathway. Free Radic. Biol. Med..

[50] Vandanmagsar B., Warfel J.D., Wicks S.E., Ghosh S., Salbaum J.M., Burk D., Dubuisson O.S., Mendoza T.M., Zhang J., Noland R.C. (2016). Impaired Mitochondrial Fat Oxidation Induces FGF21 in Muscle. Cell Rep..

[51] Haywood N.J., Slater T.A., Matthews C.J., Wheatcroft S.B. (2019). The insulin like growth factor and binding protein family: Novel therapeutic targets in obesity & diabetes. Mol. Metab..

[52] Junnila R.K., List E.O., Berryman D.E., Murrey J.W., Kopchick J.J. (2013). The GH/IGF-1 axis in ageing and longevity. Nat. Rev. Endocrinol..

[53] Lapierre L.R., Hansen M. (2012). Lessons from C. elegans: Signaling pathways for longevity. Trends Endocrinol. Metab..

[54] Vitale G., Pellegrino G., Vollery M., Hofland L.J. (2019). ROLE of IGF-1 System in the Modulation of Longevity: Controversies and New Insights From a Centenarians’ Perspective. Front. Endocrinol..

[55] Yuan R., Tsaih S.-W., Petkova S.B., De Evsikova C.M., Xing S., Marion M.A., Bogue M.A., Mills K.D., Peters L.L., Bult C.J. (2009). Aging in inbred strains of mice: Study design and interim report on median lifespans and circulating IGF1 levels. Aging Cell.

[56] Lopez-Otin C., Blasco M.A., Partridge L., Serrano M., Kroemer G. (2013). The hallmarks of aging. Cell.

[57] Milman S., Atzmon G., Huffman D.M., Wan J., Crandall J.P., Cohen P., Barzilai N. (2014). Low insulin-like growth factor-1 level predicts survival in humans with exceptional longevity. Aging Cell.

[58] Pollak M. (2012). The insulin and insulin-like growth factor receptor family in neoplasia: An update. Nat. Rev. Cancer.

[59] Villela T.R., Freire B.L., Braga N.T.P., Arantes R.R., Funari M.F.A., Alexander J.A.L., Silva I.N. (2020). Growth Hormone insensitivity (Laron syndrome): Report of a new family and review of Brazilian patients. Genet. Mol. Biol..

[60] Laron Z., Kauli R. (2016). Fifty seven years of follow-up of the Israeli cohort of Laron Syndrome patients-From discovery to treatment. Growth Horm. IGF Res..

[61] Laron Z. (2016). Epilogue: The future of Laron syndrome—The need for changes. Growth Horm. IGF Res..

[62] Janecka A., Kolodziej-Rzepa M., Biesaga B. (2016). Clinical and Molecular Features of Laron Syndrome, A Genetic Disorder Protecting from Cancer. In Vivo.

[63] Guevara-Aguirre J., Balasubramanian P., Guevara-Aguirre M., Wei M., Madia F., Cheng C.W., Hwang D., Martin-Montalvo A., Saavedra J., Ingles S. (2011). Growth hormone receptor deficiency is associated with a major reduction in pro-aging signaling, cancer, and diabetes in humans. Sci. Transl. Med..

[64] Vitale G., Barbieri M., Kamenetskaya M., Paolisso G. (2017). GH/IGF-I/insulin system in centenarians. Mech. Ageing Dev..

[65] Arai Y., Takayama M., Gondo Y., Inagaki H., Yamamura K., Nakazawa S., Kojima T., Ebihara Y., Shimizu K., Masui Y. (2008). Adipose Endocrine Function, Insulin-Like Growth Factor-1 Axis, and Exceptional Survival Beyond 100 Years of Age. J. Gerontol. Ser. A Biol. Sci. Med. Sci..

[66] Paolisso G., Ammendola S., Del Buono A., Gambardella A., Riondino M., Tagliamonte M.R., Rizzo M.R., Carella C., Varricchio M. (1997). Serum Levels of Insulin-Like Growth Factor-I (IGF-I) and IGF-Binding Protein-3 in Healthy Centenarians: Relationship with Plasma Leptin and Lipid Concentrations, Insulin Action, and Cognitive Function. J. Clin. Endocrinol. Metab..

[67] Lytras A., Tolis G. (2007). Assessment of endocrine and nutritional status in age-related catabolic states of muscle and bone. Curr. Opin. Clin. Nutr. Metab. Care.

[68] Kamei Y., Hatazawa Y., Uchitomi R., Yoshimura R., Miura S. (2020). Regulation of Skeletal Muscle Function by Amino Acids. Nutrients.

[69] Blagosklonny M.V. (2014). Calorie restriction: Decelerating mTOR-driven aging from cells to organisms (including humans). Cell Cycle.

[70] Johnson S.C., Rabinovitch P.S., Kaeberlein M. (2013). mTOR is a key modulator of ageing and age-related disease. Nature.

[71] Weichhart T. (2018). mTOR as Regulator of Lifespan, Aging, and Cellular Senescence: A Mini-Review. Gerontology.

[72] Wipperman M.F., Montrose D.C., Gotto A.M., Hajjar D.P. (2019). Mammalian Target of Rapamycin: A Metabolic Rheostat for Regulating Adipose Tissue Function and Cardiovascular Health. Am. J. Pathol..

[73] Guertin D.A., Stevens D.M., Thoreen C.C., Burds A.A., Kalaany N.Y., Moffat J., Brown M., Fitzgerald K.J., Sabatini D.M. (2006). Ablation in Mice of the mTORC Components raptor, rictor, or mLST8 Reveals that mTORC2 Is Required for Signaling to Akt-FOXO and PKCα, but Not S6K1. Dev. Cell.

[74] Papadopoli D., Boulay K., Kazak L., Pollak M., Mallette F., Topisirovic I., Hulea L. (2019). mTOR as a central regulator of lifespan and aging. F1000Research.

[75] Templeman N.M., Murphy C.T. (2018). Regulation of reproduction and longevity by nutrient-sensing pathways. J. Cell Biol..

[76] Simpson S.J., Raubenheimer D. (2009). Macronutrient balance and lifespan. Aging.

[77] Lushchak O., Strilbytska O., Piskovatska V., Storey K.B., Koliada A., Vaiserman A. (2017). The role of the TOR pathway in mediating the link between nutrition and longevity. Mech. Ageing Dev..

[78] Fontana L., Partridge L., Longo V.D. (2010). Extending healthy life span—From yeast to humans. Science.

[79] Albert V., Hall M.N. (2015). mTOR signaling in cellular and organismal energetics. Curr. Opin. Cell Biol..

[80] Kitada M., Ogura Y., Monno I., Koya D. (2019). The impact of dietary protein intake on longevity and metabolic health. EBioMedicine.

[81] Chen C.C., Jeon S.M., Bhaskar P.T., Nogueira V., Sundararajan D., Tonic I., Park Y., Hay N. (2010). FoxOs inhibit mTORC1 and activate Akt by inducing the expression of Sestrin3 and Rictor. Dev. Cell.

[82] Jiang Y., Yan F., Feng Z., Lazarovici P., Zheng W. (2019). Signaling Network of Forkhead Family of Transcription Factors (FOXO) in Dietary Restriction. Cells.

[83] Morris B.J., Willcox D.C., Donlon T.A., Willcox B.J. (2015). FOXO3: A Major Gene for Human Longevity—A Mini-Review. Gerontology.

[84] Chin R.M., Fu X., Pai M.Y., Vergnes L., Hwang H., Deng G., Diep S., Lomenick B., Meli V.S., Monsalve G.C. (2014). The metabolite alpha-ketoglutarate extends lifespan by inhibiting ATP synthase and TOR. Nature.

[85] Smith R.L., Soeters M.R., Wust R.C.I., Houtkooper R.H. (2018). Metabolic Flexibility as an Adaptation to Energy Resources and Requirements in Health and Disease. Endocr. Rev..

[86] Palacios O.M., Carmona J.J., Michan S., Chen K.Y., Manabe Y., Ward J.L., Goodyear L.J., Tong Q. (2009). Diet and exercise signals regulate SIRT3 and activate AMPK and PGC-1alpha in skeletal muscle. Aging.

[87] Canto C., Gerhart-Hines Z., Feige J.N., Lagouge M., Noriega L., Milne J.C., Elliott P.J., Puigserver P., Auwerx J. (2009). AMPK regulates energy expenditure by modulating NAD+ metabolism and SIRT1 activity. Nature.

[88] Passariello C.L., Zini M., Nassi P.A., Pignatti C., Stefanelli C. (2011). Upregulation of SIRT1 deacetylase in phenylephrine-treated cardiomyoblasts. Biochem. Biophys. Res. Commun..

[89] Lopez-Lluch G., Navas P. (2016). Calorie restriction as an intervention in ageing. J. Physiol..

[90] Jager S., Handschin C., St-Pierre J., Spiegelman B.M. (2007). AMP-activated protein kinase (AMPK) action in skeletal muscle via direct phosphorylation of PGC-1alpha. Proc. Natl. Acad. Sci. USA.

[91] Amat R., Planavila A., Chen S.L., Iglesias R., Giralt M., Villarroya F. (2009). SIRT1 controls the transcription of the peroxisome proliferator-activated receptor-gamma Co-activator-1alpha (PGC-1alpha) gene in skeletal muscle through the PGC-1alpha autoregulatory loop and interaction with MyoD. J. Biol. Chem..

[92] Terada S., Goto M., Kato M., Kawanaka K., Shimokawa T., Tabata I. (2002). Effects of low-intensity prolonged exercise on PGC-1 mRNA expression in rat epitrochlearis muscle. Biochem. Biophys. Res. Commun..

[93] Suwa M., Nakano H., Kumagai S. (2003). Effects of chronic AICAR treatment on fiber composition, enzyme activity, UCP3, and PGC-1 in rat muscles. J. Appl. Physiol..

[94] Boutant M., Cantó C. (2014). SIRT1 metabolic actions: Integrating recent advances from mouse models. Mol. Metab..

[95] Oh K.J., Han H.S., Kim M.J., Koo S.H. (2013). CREB and FoxO1: Two transcription factors for the regulation of hepatic gluconeogenesis. BMB Rep..

[96] Dillon L.M., Rebelo A.P., Moraes C.T. (2012). The role of PGC-1 coactivators in aging skeletal muscle and heart. IUBMB Life.

[97] Huh J.Y. (2018). The role of exercise-induced myokines in regulating metabolism. Arch. Pharm. Res..

[98] Cheng C.F., Ku H.C., Lin H. (2018). PGC-1alpha as a Pivotal Factor in Lipid and Metabolic Regulation. Int. J. Mol. Sci..

[99] Green C.L., Lamming D.W. (2019). Regulation of metabolic health by essential dietary amino acids. Mech. Ageing Dev..

[100] Brandhorst S., Longo V.D. (2019). Protein Quantity and Source, Fasting-Mimicking Diets, and Longevity. Adv. Nutr..

[101] Jin J., Zhang T. (2013). Effects of Glucose Restriction on Replicative Senescence of Human Diploid Fibroblasts IMR-90. Cell. Physiol. Biochem..

[102] Dutcher S.K., Roux A.E., Leroux A., Alaamery M.A., Hoffman C.S., Chartrand P., Ferbeyre G., Rokeach L.A. (2009). Pro-Aging Effects of Glucose Signaling through a G Protein-Coupled Glucose Receptor in Fission Yeast. PLoS Genet..

[103] Weinberger M., Mesquita A., Carroll T., Marks L., Yang H., Zhang Z., Ludovico P., Burhans W.C. (2010). Growth signaling promotes chronological aging in budding yeast by inducing superoxide anions that inhibit quiescence. Aging.

[104] Lee S.-J., Murphy C.T., Kenyon C. (2009). Glucose Shortens the Life Span of C. elegans by Downregulating DAF-16/FOXO Activity and Aquaporin Gene Expression. Cell Metab..

[105] Schulz T.J., Zarse K., Voigt A., Urban N., Birringer M., Ristow M. (2007). Glucose Restriction Extends Caenorhabditis elegans Life Span by Inducing Mitochondrial Respiration and Increasing Oxidative Stress. Cell Metab..

[106] Kim S.G., Buel G.R., Blenis J. (2013). Nutrient regulation of the mTOR Complex 1 signaling pathway. Mol. Cells.

[107] Bröer S., Bröer A. (2017). Amino acid homeostasis and signalling in mammalian cells and organisms. Biochem. J..

[108] Gu X., Orozco J.M., Saxton R.A., Condon K.J., Liu G.Y., Krawczyk P.A., Scaria S.M., Harper J.W., Gygi S.P., Sabatini D.M. (2017). SAMTOR is an S-adenosylmethionine sensor for the mTORC1 pathway. Science.

[109] Sangüesa G., Roglans N., Baena M., Velázquez A., Laguna J., Alegret M. (2019). mTOR is a Key Protein Involved in the Metabolic Effects of Simple Sugars. Int. J. Mol. Sci..

[110] Yasuda M., Tanaka Y., Kume S., Morita Y., Chin-Kanasaki M., Araki H., Isshiki K., Araki S.-i., Koya D., Haneda M. (2014). Fatty acids are novel nutrient factors to regulate mTORC1 lysosomal localization and apoptosis in podocytes. Biochim. Biophys. Acta.

[111] Chaumontet C., Azzout-Marniche D., Blais A., Piedcoq J., Tomé D., Gaudichon C., Even P.C. (2019). Low-protein and methionine, high-starch diets increase energy intake and expenditure, increase FGF21, decrease IGF-1, and have little effect on adiposity in mice. Am. J. Physiol. Regul. Integr. Comp. Physiol..

[112] Inagaki T., Lin V.Y., Goetz R., Mohammadi M., Mangelsdorf D.J., Kliewer S.A. (2008). Inhibition of Growth Hormone Signaling by the Fasting-Induced Hormone FGF21. Cell Metab..

[113] Mair W., Dillin A. (2008). Aging and survival: The genetics of life span extension by dietary restriction. Annu. Rev. Biochem..

[114] Lee C., Longo V. (2016). Dietary restriction with and without caloric restriction for healthy aging. F1000Research.

[115] Fontana L., Weiss E.P., Villareal D.T., Klein S., Holloszy J.O. (2008). Long-term effects of calorie or protein restriction on serum IGF-1 and IGFBP-3 concentration in humans. Aging Cell.

[116] Stekovic S., Hofer S.J., Tripolt N., Aon M.A., Royer P., Pein L., Stadler J.T., Pendl T., Prietl B., Url J. (2019). Alternate Day Fasting Improves Physiological and Molecular Markers of Aging in Healthy, Non-obese Humans. Cell Metab..

[117] Most J., Tosti V., Redman L.M., Fontana L. (2017). Calorie restriction in humans: An update. Ageing Res. Rev..

[118] Hansen M., Chandra A., Mitic L.L., Onken B., Driscoll M., Kenyon C. (2008). A role for autophagy in the extension of lifespan by dietary restriction in C. elegans. PLoS Genet..

[119] Santos J., Leitao-Correia F., Sousa M.J., Leao C. (2016). Dietary Restriction and Nutrient Balance in Aging. Oxid. Med. Cell Longev..

[120] Gonzalez-Freire M., Diaz-Ruiz A., Hauser D., Martinez-Romero J., Ferrucci L., Bernier M., de Cabo R. (2020). The road ahead for health and lifespan interventions. Ageing Res. Rev..

[121] Riera C.E., Merkwirth C., De Magalhaes Filho C.D., Dillin A. (2016). Signaling Networks Determining Life Span. Annu. Rev. Biochem..

[122] Mattison J.A., Colman R.J., Beasley T.M., Allison D.B., Kemnitz J.W., Roth G.S., Ingram D.K., Weindruch R., de Cabo R., Anderson R.M. (2017). Caloric restriction improves health and survival of rhesus monkeys. Nat. Commun..

[123] Shintani H., Shintani T., Ashida H., Sato M. (2018). Calorie Restriction Mimetics: Upstream-Type Compounds for Modulating Glucose Metabolism. Nutrients.

[124] Weimer S., Priebs J., Kuhlow D., Groth M., Priebe S., Mansfeld J., Merry T.L., Dubuis S., Laube B., Pfeiffer A.F. (2014). D-Glucosamine supplementation extends life span of nematodes and of ageing mice. Nat. Commun..

[125] Ingram D.K., Roth G.S. (2015). Calorie restriction mimetics: Can you have your cake and eat it, too?. Ageing Res. Rev..

[126] Lamming D.W., Ye L., Sabatini D.M., Baur J.A. (2013). Rapalogs and mTOR inhibitors as anti-aging therapeutics. J. Clin. Investig..

[127] Sinclair D.A., Guarente L. (2014). Small-Molecule Allosteric Activators of Sirtuins. Annu. Rev. Pharmacol. Toxicol..

[128] Martens C.R., Denman B.A., Mazzo M.R., Armstrong M.L., Reisdorph N., McQueen M.B., Chonchol M., Seals D.R. (2018). Chronic nicotinamide riboside supplementation is well-tolerated and elevates NAD+ in healthy middle-aged and older adults. Nat. Commun..

[129] Minois N. (2014). Molecular Basis of the ‘Anti-Aging’ Effect of Spermidine and Other Natural Polyamines—A Mini-Review. Gerontology.

[130] Schwarz C., Stekovic S., Wirth M., Benson G., Royer P., Sigrist S.J., Pieber T., Dammbrueck C., Magnes C., Eisenberg T. (2018). Safety and tolerability of spermidine supplementation in mice and older adults with subjective cognitive decline. Aging.

[131] Eisenberg T., Abdellatif M., Schroeder S., Primessnig U., Stekovic S., Pendl T., Harger A., Schipke J., Zimmermann A., Schmidt A. (2016). Cardioprotection and lifespan extension by the natural polyamine spermidine. Nat. Med..

[132] D’Adamo S., Cetrullo S., Guidotti S., Silvestri Y., Minguzzi M., Santi S., Cattini L., Filardo G., Flamigni F., Borzì R.M. (2020). Spermidine rescues the deregulated autophagic response to oxidative stress of osteoarthritic chondrocytes. Free Radic. Biol. Med..

[133] Madeo F., Eisenberg T., Pietrocola F., Kroemer G. (2018). Spermidine in health and disease. Science.

[134] Madeo F., Carmona-Gutierrez D., Hofer S.J., Kroemer G. (2019). Caloric Restriction Mimetics against Age-Associated Disease: Targets, Mechanisms, and Therapeutic Potential. Cell Metab..

[135] Solon-Biet S.M., Mitchell S.J., de Cabo R., Raubenheimer D., Le Couteur D.G., Simpson S.J. (2015). Macronutrients and caloric intake in health and longevity. J. Endocrinol..

[136] Westerterp-Plantenga M.S., Nieuwenhuizen A., Tome D., Soenen S., Westerterp K.R. (2009). Dietary protein, weight loss, and weight maintenance. Annu. Rev. Nutr..

[137] van den Brandt P.A. (2019). Red meat, processed meat, and other dietary protein sources and risk of overall and cause-specific mortality in The Netherlands Cohort Study. Eur. J. Epidemiol..

[138] Etemadi A., Sinha R., Ward M.H., Graubard B.I., Inoue-Choi M., Dawsey S.M., Abnet C.C. (2017). Mortality from different causes associated with meat, heme iron, nitrates, and nitrites in the NIH-AARP Diet and Health Study: Population based cohort study. Br. Med. J..

[139] Song M., Fung T.T., Hu F.B., Willett W.C., Longo V.D., Chan A.T., Giovannucci E.L. (2016). Association of Animal and Plant Protein Intake With All-Cause and Cause-Specific Mortality. JAMA Intern. Med..

[140] Huang J., Liao L.M., Weinstein S.J., Sinha R., Graubard B.I., Albanes D. (2020). Association Between Plant and Animal Protein Intake and Overall and Cause-Specific Mortality. JAMA Intern. Med..

[141] Fontana L., Cummings N.E., Arriola Apelo S.I., Neuman J.C., Kasza I., Schmidt B.A., Cava E., Spelta F., Tosti V., Syed F.A. (2016). Decreased Consumption of Branched-Chain Amino Acids Improves Metabolic Health. Cell Rep..

[142] Brown-Borg H.M., Buffenstein R. (2017). Cutting back on the essentials: Can manipulating intake of specific amino acids modulate health and lifespan?. Ageing Res. Rev..

[143] Lee B.C., Kaya A., Gladyshev V.N. (2016). Methionine restriction and life-span control. Ann. N. Y. Acad. Sci..

[144] Malloy V.L., Krajcik R.A., Bailey S.J., Hristopoulos G., Plummer J.D., Orentreich N. (2006). Methionine restriction decreases visceral fat mass and preserves insulin action in aging male Fischer 344 rats independent of energy restriction. Aging Cell.

[145] Elshorbagy A.K., Valdivia-Garcia M., Mattocks D.A., Plummer J.D., Smith A.D., Drevon C.A., Refsum H., Perrone C.E. (2011). Cysteine supplementation reverses methionine restriction effects on rat adiposity: Significance of stearoyl-coenzyme A desaturase. J. Lipid Res..

[146] Perrone C.E., Mattocks D.A., Hristopoulos G., Plummer J.D., Krajcik R.A., Orentreich N. (2008). Methionine restriction effects on 11 -HSD1 activity and lipogenic/lipolytic balance in F344 rat adipose tissue. J. Lipid Res..

[147] Ables G.P., Perrone C.E., Orentreich D., Orentreich N. (2012). Methionine-restricted C57BL/6J mice are resistant to diet-induced obesity and insulin resistance but have low bone density. PLoS ONE.

[148] Wanders D., Forney L.A., Stone K.P., Burk D.H., Pierse A., Gettys T.W. (2017). FGF21 Mediates the Thermogenic and Insulin-Sensitizing Effects of Dietary Methionine Restriction but Not Its Effects on Hepatic Lipid Metabolism. Diabetes.

[149] Mirzaei H., Suarez J.A., Longo V.D. (2014). Protein and amino acid restriction, aging and disease: From yeast to humans. Trends Endocrinol. Metab..

[150] Zapata R.C., Singh A., Ajdari N.M., Chelikani P.K. (2018). Dietary Tryptophan Restriction Dose-Dependently Modulates Energy Balance, Gut Hormones, and Microbiota in Obesity-Prone Rats. Obesity.

[151] Xiao F., Huang Z., Li H., Yu J., Wang C., Chen S., Meng Q., Cheng Y., Gao X., Li J. (2011). Leucine deprivation increases hepatic insulin sensitivity via GCN2/mTOR/S6K1 and AMPK pathways. Diabetes.

[152] Takenaka A., Oki N., Takahashi S.I., Noguchi T. (2000). Dietary restriction of single essential amino acids reduces plasma insulin-like growth factor-I (IGF-I) but does not affect plasma IGF-binding protein-1 in rats. J. Nutr..

[153] Strasser B., Volaklis K., Fuchs D., Burtscher M. (2018). Role of Dietary Protein and Muscular Fitness on Longevity and Aging. Aging Dis..

[154] Balasubramanian P., Longo V.D. (2016). Growth factors, aging and age-related diseases. Growth Horm. IGF Res..

[155] Levine M.E., Suarez J.A., Brandhorst S., Balasubramanian P., Cheng C.W., Madia F., Fontana L., Mirisola M.G., Guevara-Aguirre J., Wan J. (2014). Low protein intake is associated with a major reduction in IGF-1, cancer, and overall mortality in the 65 and younger but not older population. Cell Metab..

[156] Naghshi S., Sadeghi O., Willett W.C., Esmaillzadeh A. (2020). Dietary intake of total, animal, and plant proteins and risk of all cause, cardiovascular, and cancer mortality: Systematic review and dose-response meta-analysis of prospective cohort studies. Br. Med. J..

[157] Bell J.T., Spector T.D. (2011). A twin approach to unraveling epigenetics. Trends Genet..

[158] Gadecka A., Bielak-Zmijewska A. (2019). Slowing Down Ageing: The Role of Nutrients and Microbiota in Modulation of the Epigenome. Nutrients.

[159] Rinninella E., Cintoni M., Raoul P., Lopetuso L.R., Scaldaferri F., Pulcini G., Miggiano G.A.D., Gasbarrini A., Mele M.C. (2019). Food Components and Dietary Habits: Keys for a Healthy Gut Microbiota Composition. Nutrients.

[160] Lees-Murdock D.J., Walsh C.P., Dollin C., Hughes C.F., McNulty H., Strain J.J., Ward M., Amenyah S.D. (2020). Nutritional Epigenomics and Age-Related Disease. Curr. Dev. Nutr..

[161] McKay J.A., Mathers J.C. (2011). Diet induced epigenetic changes and their implications for health. Acta Physiol..

[162] Kok D.E.G., Dhonukshe-Rutten R.A.M., Lute C., Heil S.G., Uitterlinden A.G., van der Velde N., van Meurs J.B.J., van Schoor N.M., Hooiveld G.J.E.J., de Groot L.C.P.G.M. (2015). The effects of long-term daily folic acid and vitamin B12 supplementation on genome-wide DNA methylation in elderly subjects. Clin. Epigenetics.

[163] Chamberlain J.A., Dugué P.-A., Bassett J.K., Hodge A.M., Brinkman M.T., Joo J.E., Jung C.-H., Makalic E., Schmidt D.F., Hopper J.L. (2018). Dietary intake of one-carbon metabolism nutrients and DNA methylation in peripheral blood. Am. J. Clin. Nutr..

[164] Metes-Kosik N., Luptak I., DiBello P.M., Handy D.E., Tang S.-S., Zhi H., Qin F., Jacobsen D.W., Loscalzo J., Joseph J. (2012). Both selenium deficiency and modest selenium supplementation lead to myocardial fibrosis in mice via effects on redox-methylation balance. Mol. Nutr. Food Res..

[165] Nandakumar V., Vaid M., Katiyar S.K. (2011). (-)-Epigallocatechin-3-gallate reactivates silenced tumor suppressor genes, Cip1/p21 and p16INK4a, by reducing DNA methylation and increasing histones acetylation in human skin cancer cells. Carcinogenesis.

[166] Zhao Z., Shilatifard A. (2019). Epigenetic modifications of histones in cancer. Genome Biol..

[167] Bouras T., Fu M., Sauve A.A., Wang F., Quong A.A., Perkins N.D., Hay R.T., Gu W., Pestell R.G. (2005). SIRT1 Deacetylation and Repression of p300 Involves Lysine Residues 1020/1024 within the Cell Cycle Regulatory Domain 1. J. Biol. Chem..

[168] Cetrullo S., D’Adamo S., Tantini B., Borzi R.M., Flamigni F. (2015). mTOR, AMPK, and Sirt1: Key Players in Metabolic Stress Management. Crit. Rev. Eukaryot. Gene Expr..

[169] Boccardi V., Paolisso G., Mecocci P. (2016). Nutrition and lifestyle in healthy aging: The telomerase challenge. Aging.

[170] Davinelli S., Trichopoulou A., Corbi G., De Vivo I., Scapagnini G. (2019). The potential nutrigeroprotective role of Mediterranean diet and its functional components on telomere length dynamics. Ageing Res. Rev..

[171] García-Calzón S., Moleres A., Martínez-González M.A., Martínez J.A., Zalba G., Marti A. (2015). Dietary total antioxidant capacity is associated with leukocyte telomere length in a children and adolescent population. Clin. Nutr..

[172] Wang L., Sinnott-Armstrong N., Wagschal A., Wark A.R., Camporez J.-P., Perry R.J., Ji F., Sohn Y., Oh J., Wu S. (2020). A MicroRNA Linking Human Positive Selection and Metabolic Disorders. Cell.

[173] Slattery M., Herrick J., Mullany L., Stevens J., Wolff R. (2016). Diet and lifestyle factors associated with miRNA expression in colorectal tissue. Pharm. Pers. Med..

[174] Davis C.D., Ross S.A. (2008). Evidence for dietary regulation of microRNA expression in cancer cells. Nutr. Rev..

[175] Lançon A., Michaille J.-J., Latruffe N. (2013). Effects of dietary phytophenols on the expression of microRNAs involved in mammalian cell homeostasis. J. Sci. Food Agric..

[176] Campbell K. (2020). Do the microRNAs we eat affect gene expression?. Nature.

[177] Medina-Remón A., Kirwan R., Lamuela-Raventós R.M., Estruch R. (2017). Dietary patterns and the risk of obesity, type 2 diabetes mellitus, cardiovascular diseases, asthma, and neurodegenerative diseases. Crit. Rev. Food Sci. Nutr..

[178] Buettner D., Skemp S. (2016). Blue Zones. Am. J. Lifestyle Med..

[179] Rizzuto D., Fratiglioni L. (2014). Lifestyle Factors Related to Mortality and Survival: A Mini-Review. Gerontology.

[180] Pignolo R.J. (2019). Exceptional Human Longevity. Mayo Clin. Proc..

[181] Willcox B.J., Willcox D.C. (2013). Caloric restriction, caloric restriction mimetics, and healthy aging in Okinawa. Curr. Opin. Clin. Nutr. Metab. Care.

[182] Vasto S., Buscemi S., Barera A., Di Carlo M., Accardi G., Caruso C. (2014). Mediterranean Diet and Healthy Ageing: A Sicilian Perspective. Gerontology.

[183] Del Bo C., Marino M., Martini D., Tucci M., Ciappellano S., Riso P., Porrini M. (2019). Overview of Human Intervention Studies Evaluating the Impact of the Mediterranean Diet on Markers of DNA Damage. Nutrients.

[184] Ros M., Carrascosa J.M. (2020). Current nutritional and pharmacological anti-aging interventions. Biochim. Biophys. Acta.

[185] Peyrol J., Riva C., Amiot M. (2017). Hydroxytyrosol in the Prevention of the Metabolic Syndrome and Related Disorders. Nutrients.

[186] Angeloni C., Malaguti M., Barbalace M., Hrelia S. (2017). Bioactivity of Olive Oil Phenols in Neuroprotection. Int. J. Mol. Sci..

[187] Facchini A., Cetrullo S., D’Adamo S., Guidotti S., Minguzzi M., Facchini A., Borzi R.M., Flamigni F. (2014). Hydroxytyrosol prevents increase of osteoarthritis markers in human chondrocytes treated with hydrogen peroxide or growth-related oncogene alpha. PLoS ONE.

[188] Cetrullo S., D’Adamo S., Guidotti S., Borzi R.M., Flamigni F. (2016). Hydroxytyrosol prevents chondrocyte death under oxidative stress by inducing autophagy through sirtuin 1-dependent and -independent mechanisms. Biochim. Biophys. Acta.

[189] Sofi F., Macchi C., Abbate R., Gensini G.F., Casini A. (2013). Mediterranean diet and health. BioFactors.

[190] Estruch R. (2010). Anti-inflammatory effects of the Mediterranean diet: The experience of the PREDIMED study. Proc. Nutr. Soc..

[191] Schwingshackl L., Morze J., Hoffmann G. (2019). Mediterranean diet and health status: Active ingredients and pharmacological mechanisms. Br. J. Pharmacol..

[192] Martel J., Ojcius D.M., Ko Y.-F., Ke P.-Y., Wu C.-Y., Peng H.-H., Young J.D. (2019). Hormetic Effects of Phytochemicals on Health and Longevity. Trends Endocrinol. Metab..

[193] Soultoukis G.A., Partridge L. (2016). Dietary Protein, Metabolism, and Aging. Annu. Rev. Biochem..

[194] Shim H.S., Longo V.D. (2015). A protein restriction-dependent sulfur code for longevity. Cell.

[195] Lynch C.J., Adams S.H. (2014). Branched-chain amino acids in metabolic signalling and insulin resistance. Nat. Rev. Endocrinol..

[196] Waern R.V., Cumming R.G., Blyth F., Naganathan V., Allman-Farinelli M., Le Couteur D., Simpson S.J., Kendig H., Hirani V. (2015). Adequacy of nutritional intake among older men living in Sydney, Australia: Findings from the Concord Health and Ageing in Men Project (CHAMP). Br. J. Nutr..

[197] Le Couteur D.G., Solon-Biet S., Cogger V.C., Mitchell S.J., Senior A., de Cabo R., Raubenheimer D., Simpson S.J. (2016). The impact of low-protein high-carbohydrate diets on aging and lifespan. Cell Mol. Life Sci..

[198] Liao C.-Y., Rikke B.A., Johnson T.E., Diaz V., Nelson J.F. (2010). Genetic variation in the murine lifespan response to dietary restriction: From life extension to life shortening. Aging Cell.

[199] Murphy C.T., Perez-Matos M.C., Mair W.B. (2020). Predicting longevity responses to dietary restriction: A stepping stone toward precision geroscience. PLoS Genet..

[200] Murphy C.T., Jin K., Wilson K.A., Beck J.N., Nelson C.S., Brownridge G.W., Harrison B.R., Djukovic D., Raftery D., Brem R.B. (2020). Genetic and metabolomic architecture of variation in diet restriction-mediated lifespan extension in Drosophila. PLoS Genet..

